# Sustained microglial activation in the area postrema of collagen-induced arthritis mice

**DOI:** 10.1186/s13075-021-02657-x

**Published:** 2021-10-29

**Authors:** Takayuki Matsushita, Kazuhiro Otani, Yohsuke Oto, Yukari Takahashi, Daitaro Kurosaka, Fusao Kato

**Affiliations:** 1 Division of Rheumatology, Department of Internal Medicine, The Jikei University School of Medicine, 3-25-8 Nishi-shimbashi, Minato-ku, Tokyo, 105-8461 Japan; 2 Department of Neuroscience, The Jikei University School of Medicine, 3-25-8 Nishi-shimbashi, Minato-ku, Tokyo, Japan; 3 Center for Neuroscience of Pain, The Jikei University School of Medicine, 3-25-8 Nishi-shimbashi, Minato-ku, Tokyo, Japan

**Keywords:** Central nervous system, Circumventricular organs, Area postrema, Microglia, Interleukin-1β, Collagen-induced arthritis, Rheumatoid arthritis

## Abstract

**Background:**

Central nervous system (CNS)-mediated symptoms, such as fatigue, depression, and hyperalgesia, are common complications among patients with rheumatoid arthritis (RA). However, it remains unclear how the peripheral pathology of RA spreads to the brain. Accumulated evidence showing an association between serum cytokine levels and aberrant CNS function suggests that humoral factors participate in this mechanism. In contrast to the well-known early responses of microglia (CNS-resident immune cells) in the area postrema [AP; a brain region lacking a blood–brain barrier (BBB)] to experimental inflammation, microglial alterations in the AP during chronic inflammation like RA remain unclear. Therefore, to determine whether microglia in the AP can react to persistent autoimmune-arthritis conditions, we analyzed these cells in a mouse model of collagen-induced arthritis (CIA).

**Methods:**

Microglial number and morphology were analyzed in the AP of CIA and control mice (administered Freund’s adjuvant or saline). Immunostaining for ionized calcium-binding adaptor molecule-1 was performed at various disease phases: “pre-onset” [post-immunization day (PID) 21], “establishment” (PID 35), and “chronic” (PID 56 and 84). Quantitative analyses of microglial number and morphology were performed, with principal component analysis used to classify microglia. Interleukin-1β (IL-1β) mRNA expression was analyzed by multiple fluorescent in situ hybridization and real-time polymerase chain reaction. Behavioral changes were assessed by sucrose preference test.

**Results:**

Microglia in the AP significantly increased in density and exhibited changes in morphology during the establishment and chronic phases, but not the pre-onset phase. Non-subjective clustering classification of cell morphology (CIA, 1,256 cells; saline, 852 cells) showed that the proportion of highly activated microglia increased in the CIA group during establishment and chronic phases. Moreover, the density of IL-1β-positive microglia, a hallmark of functional activation, was increased in the AP. Sucrose preferences in CIA mice negatively correlated with IL-1β expression in brain regions containing the AP.

**Conclusions:**

Our findings demonstrate that microglia in the AP can sustain their activated state during persistent autoimmune arthritis, which suggests that chronic inflammation, such as RA, may affect microglia in brain regions lacking a BBB and have various neural consequences.

**Supplementary Information:**

The online version contains supplementary material available at 10.1186/s13075-021-02657-x.

## Background

Rheumatoid arthritis (RA) is frequently accompanied by symptoms indicative of changes in central nervous system (CNS) functions [1], such as depression, fatigue, sleep disturbance, and hyperalgesia [2–5]. Recently accumulated evidence indicates that such CNS-mediated symptoms remain even after the mitigation of principal polyarthritis pathology with advanced pharmacological therapeutic strategies, such as biologics [6], making these CNS symptoms a primary concern for treatment of RA [7]. The long-lasting influence of RA likely results from mechanisms enabling sustained peripheral immune information to affect the CNS. These mechanisms include direct activation of CNS cells via humoral factors and afferent nerve-mediated transfer of immune information to the brain, such as those transmitted via the vagus nerve [8] and sensory autonomic nervous system [9]. Additionally, and with particular consideration for the aftereffects of long-lasting RA, it is expected that even short-term activation of autoimmune activity would promote plastic adaptation of the CNS, which may result in sustained CNS-mediated complications, such as chronic pain [10]. Despite lines of evidence indicating a close association between the disease activity of RA and neuropsychological symptoms [1, 11, 12], the underlying cellular mechanisms remain only poorly explored.

Preclinical studies using animal models of RA are the gold-standard strategy for identifying molecular and cellular mechanisms underlying RA. Also in frequently used rodent RA models, morphofunctional alterations in CNS have been documented. For example, mice with antibody-induced arthritis exhibited astrocyte and microglial activation in the lumber dorsal horn 29 days after antibody injection [13]. In addition, rats with adjuvant-induced arthritis exhibited increased expression of c-fos, a marker for neuronal activation, in the lumber spinal-dorsal horn 21 days after injection [14]. As these spinal regions are sites targeted by primary sensory fibers, these reports support the interpretation that afferent nerves mediate the peripheral-to-CNS immune signaling observed with RA. In contrast, rats with collagen-induced arthritis (CIA) showed significantly higher distributions of intravenously injected fluorescein in the cortex and hippocampus compared with saline-injected rats 17 days after the first immunization [15]. The latter report favors augmented direct humoral channeling from the periphery to CNS at a relatively early period of RA progression in arthritis models. Generally, such a direct humoral channel would play essential roles in early and immediate signaling of circulating pathological information to the CNS, such as responses observed a few hours after injections of cytokines or lipopolysaccharide (LPS) [16–18]. However, it remains unknown whether such direct humoral influences persist in long-lasting RA to continuously influence the activity of cells in the CNS.

To address this issue, we focused on microglia, the “frontline” immune cells of the CNS, in the area postrema (AP), one of the sensory circumventricular organs (sCVOs). Because the AP has attenuated blood–brain barrier (BBB) function, it is responsible for direct humoral signaling from the peripheral circulation to the brain tissue. To examine whether microglia show sustained morphological activation at various time points throughout the long duration of RA progression, we evaluated the morphological activation of microglia from the pre-onset phase to the chronic phase up to 84 days post-immunization in a mouse model of CIA. In addition, we examined if behavioral changes accompanied microglial activation and related cytokine expression.

## Methods

### Animals

Male DBA/1J mice were purchased from Sankyo Labo Service (Tokyo, Japan). Animals were housed in groups of 4 to 6 for periods other than those described below for behavioral tests, and maintained on a light/dark cycle of 12:12 h with food and water available ad libitum.

### Detecting the circumventricular organs using fluorescein isothiocyanate

To visualize the sCVOs of DBA/1J mice, a fluorescein isothiocyanate (FITC) method was used according to a previous report [19]. Briefly, 12-week-old mice were anesthetized using isoflurane (3% in 100% O_2_) and transcardially perfused with the following: first, 0.1 M phosphate-buffered saline (PBS), 5 ml; second, FITC in PBS (0.1 mg/ml), 25 ml; third, PBS, 12.5 ml; and finally, 4% paraformaldehyde (PFA) in 0.1 M phosphate buffer (PB), 40 ml. Dissected brains were postfixed overnight and cryoprotected. Brain blocks were embedded in OCT Compound (Sakura Finetek, Tokyo, Japan) and stored at – 80 °C. Consecutive coronal sections (20-μm thick) were obtained throughout brain regions containing the third ventricle and medulla using a cryostat (CM1850; Leica Biosystems, Tokyo, Japan). Every second section was embedded in anti-fading Aqua Poly/Mount (18606; Polysciences, Warrington, PA, USA) onto coverslips.

### Collagen-induced arthritis

After acclimation for 1 week, 7-week-old mice were used. In accordance with a previous report, the immunization procedure comprised two intradermal injections at the base of the tail on post-immunization day (PID) 0 and PID 21 [20, 21]. In the CIA group (*n* = 117), a first immunization of bovine type II collagen (200 μg/mouse; Collagen Research Center, Tokyo, Japan) dissolved in 0.1 M acetic acid (4 mg/mL) emulsified in complete Freund’s adjuvant (CFA; Becton Dickinson and Company, Franklin Lakes, NJ, USA) was administered on PID 0, with a booster immunization of bovine type II collagen dissolved in 0.1 M acetic acid emulsified in incomplete Freund’s adjuvant (IFA; Becton Dickinson and Company). In the Freund’s adjuvant group (FA group; *n* = 16), 0.1 M acetic acid without type II collagen emulsified in CFA (PID 0) and IFA (PID 21) were administered in the same manner. In the saline group (*n* = 64), an equivalent volume of saline was administered. Immunization was performed in a blinded fashion in the CIA and FA groups, but not the saline group, because of differences in appearance of the saline and emulsions. In accordance with a previous report [21], arthritis severity was determined using arthritis scores for all four limbs on the following scale: 0, normal; 1, swelling of digits alone or localized swelling of wrist and ankle joints; 2, swelling of both digits and wrist or ankle joints; and 3, swelling of a whole limb. “Total arthritis score” defined the sum of the scores for all four limbs. Brain analyses (described below) were performed on PID 21 and PID 35 and considered to represent the pre-onset and establishment phases, respectively, while those on PID 56 and PID 84 represented chronic phases. Several mice exhibiting ulceration around the anus caused by CFA (CIA, 6 of 117; FA, 1 of 16) were excluded from further analyses. Finally, 111 CIA mice, 15 FA mice, and 64 saline mice were used in this study.

### Sucrose preference test

The sucrose preference test was performed on the basis of a previous report [22]. Briefly, mice were moved from their group-housed cage to single-housed cages (i.e., one mouse per cage), which were equipped with two drinking bottles of identical appearance that were equally and freely accessible. Food was freely accessible and the temperature and humidity were identical to the group-housed cages. The pre-test session started at 10:00 on PID 28, and the two bottles were filled with either 2% sucrose (w/v) or plain water. The positions of the two bottles were interchanged at 19:00 every day, at which point the weight of each bottle (as an indirect measure of volume) was measured. Twelve hours later (i.e., at 07:00 the following day), the weights of bottles were measured to calculate the decrease as an estimation of the amount of liquid consumed during the dark phase. The sucrose preference of each animal was defined as the ratio of consumed sucrose-containing water to the total water volume consumed during the night of PID 33 (19:00 to 07:00 the next morning; on the fifth individual housing day) and PID 34. Bottle positions were not identical for PID 33 and PID 34. The measurement of water weight was performed in a blinded manner to the content of the water.

### Tissue preparation

For immunohistochemistry, mice under anesthesia were transcardially perfused with PBS followed by 4% PFA in 0.1 M PB. After post-fixation, the brain was cryoprotected. Brain blocks were embedded in OCT compound and stored at – 80 °C. Sections containing the SFO, OVLT, or AP were obtained using a cryostat at a thickness of 20 μm. For in situ hybridization, mice under anesthesia were transcardially perfused with PBS. The unfixed medulla was dissected and frozen in isopentane on dry ice, and 16-μm coronal sections were obtained using a cryostat.

### Immunohistochemistry

Sections were washed in PBS and then incubated in blocking solution containing 1% bovine serum albumin and 0.3% Triton X-100 in PBS for 1 h at room temperature. Subsequently, sections were incubated for 21 h at 4 °C with rabbit anti-mouse ionized calcium-binding adaptor molecule 1 (Iba-1) (1:4000; Wako Chemicals, Osaka, Japan), mouse anti-mouse glial fibrillary acidic protein (GFAP) (1:2000, G3893; Sigma–Aldrich, St. Louis, MO, USA), and/or American-hamster anti-mouse CD31 (1:100, 2HB; Developmental Studies of Hybridoma Bank, Iowa University, Iowa City, IA, USA). Rabbit nonspecific IgG (20 μg/μL, 5742S; Cell Signaling Technology, Danvers, MA, USA) was used as an isotype control for the anti-Iba-1 antibody. After rinsing in PBS, sections were incubated for 2 h at room temperature with the following secondary antibodies: Alexa Fluor 488-conjugated goat anti-mouse IgG (1:1000; Thermo Fisher Scientific, Rockford, IL, USA), Alexa Fluor 568-conjugated goat anti-rabbit IgG (1:1000; Thermo Fisher Scientific), and/or Alexa Fluor 647-conjugated goat anti-American-hamster IgG (1:400; Jackson ImmunoResearch, West Grove, PA, USA). Sections were then washed with PBS and incubated with 4′,6-diamidino-2-phenylindole (DAPI) (1 μg/ml; Dojindo, Kumamoto, Japan) for nuclear staining. Slices were embedded in anti-fading Aqua Poly/Mount on coverslips.

### Multiplex fluorescent in situ hybridization

Multiplex fluorescent RNAscope [Advanced Cell Diagnosis (ACD), Hayward, CA, USA; Medical & Biological Laboratories, Nagoya, Japan] was performed using probes for Iba-1 (Mm-Aif1, #319141; ACD) and IL-1β (Mm-Il1b-C2, #316891C2; ACD), in accordance with the manufacturer’s instructions. We used RNAscope probe Mm (#320881, ACD) targeting housekeeping genes, Cyclophilin B and Polr2A, for positive control and probe (#320871, ACD) for negative control. Briefly, after fixation in 10% neural-buffered formalin at 4 °C for 15 min, sections were washed with PBS, incubated in ethanol, and air-dried. Sections were incubated with protease III (diluted 1:1 with PBS) for 30 min. After additional PBS washing, probe hybridization and amplification steps were performed. Iba-1 (Alexa 488) and IL-1β (Atto 550) probe-stained sections were incubated with DAPI and mounted with Aqua Poly/Mount on coverslips.

### Image acquisition

All fluorescence images were obtained using laser-scanning confocal microscopy (FV1200; Olympus, Tokyo, Japan). Grayscale (16-bit) images were captured with a c-MOS camera (1024 × 1024 pixels, DP80; Olympus) and saved in TIFF format.

### Quantitative analysis for immunohistochemistry

All image analyses were performed by a blinded examiner using ImageJ (National Institute of Mental Health, Bethesda, MD, USA). Images for Iba-1 immunosignal were captured with a 20× objective lens to identify microglia cells using the “triangle methods” with the same threshold value for all analyses. The number of microglia was counted using the “analyze particle” function in ImageJ by setting “size (pixel^2^)” to 75 - infinity and “circularity” to 0.0–1.00. The area of sCVOs was determined using DAPI staining in each image by identifying areas with high DAPI-positive signal density. After measuring the sCVO area, the ratios of Iba-1-immunopositive area to sCVO area (in %) and specific number of microglia (in/mm^2^) were calculated. Values were calculated using the mean value of 4–5 sections from each animal except for the analysis in the saline group on PID 35, where the mean value was calculated using the mean of two sections per animal.

For evaluation of microglial morphology, quantitation was performed on immunostained images using a × 40 objective lens. Regions of interest were placed on the four main divisions of the AP based upon GFAP immunostaining, as described previously (Supplementary Figure 1) [23, 24]. Binary images in each region of interest were acquired using the same threshold algorithm (at least eight regions from two slices per mouse). To extract single cell images, binary images were segmented using the “analyze particle” function by setting “size (pixel^2^)” as 300-infinity and “circularity” as 0.0–1.00. The following twelve morphological parameters were measured in each cell: perimeter, area, ratio of perimeter to area, ferret length, minimum ferret length, maximum and minimum diameter of approximate ellipse, aspect ratio, (minimum diameter/maximum diameter), ratio of width to height, circularity, roundness, and solidity (Supplementary Figure 2).

### Quantitative analysis for multiplex fluorescent in situ hybridization

A blinded examiner performed the following image analyses. First, using the “max entropy” threshold method in ImageJ, separate binary images were created for each IL-1β and Iba-1 mRNA signals obtained after RNAscope processing. Numbers of puncta for IL-1β and Iba-1 located within DAPI-positive nuclei within the AP were counted. In accordance with the scoring guideline for RNAscope images provided by ACD, IL-1β-positive microglia cells [identified by nuclear Iba-1 and IL-1β mRNA signals (IL-1β ^+^Iba-1^+^DAPI^+^)] were classified using the following IL-1β expression scales: *“*IL-1β ^neg^Iba-1^*+*^DAPI^+^”, no-expression; “IL-1β ^low^Iba-1^+^DAPI^+^”, coexistence of 1–3 nuclear puncta; and “IL-1β ^high^Iba-1^*+*^DAPI^+^”, coexistence of four or more nuclear puncta. Values from two slices from a single mouse were averaged.

### Quantitative real-time polymerase chain reaction

For arthritis analysis, RNA extraction and real-time polymerase chain reaction (RT-PCR) were performed as previously described [21]. Briefly, total RNA was extracted from four amputated limbs using a RNeasy Lipid Tissue Mini Kit (Qiagen, Tokyo, Japan). Real-time PCR was performed using an Applied Biosystem StepOnePlus Real-Time PCR System (Thermo Fisher Scientific) with Taqman probes and the following primers: IL-1β (Mm01336189_m1), IL-6 (Mm00446190_m1), and Actb (Mm00607939_s1).

For brain analysis, RNA extraction and RT-PCR were performed as follows. After transcardial perfusion with PBS, total RNA was extracted from the dissected medulla containing of the AP (from the rostral to caudal end) using a RNeasy Lipid Tissue Mini Kit (Qiagen). mRNA extraction from the whole brain after separating the medulla oblongata was similarly performed. RT-PCR was performed as described above with the following primers: IL-1β (Mm01336189_m1), IL-6 (Mm00446190_m1), TNF-α (Mm00443258_m1), TGF-β (Mm01178820_m1), Itgam (Mm00434455_m1), and Gapdh (Mm99999915_ g1).

Expression levels normalized to *Actb* or *Gapdh* were analyzed using the ^ΔΔ^CT method. mRNA expression levels were represented as values relative to the average of the saline group.

### Statistical analysis

Data are expressed as mean ± SEM. All statistical analyses were performed using R (version 3.6.1; the R foundation for Statistical Computing, Vienna, Austria) and EZR (Saitama Medical Center, Jichi Medical University, Saitama, Japan) [25]. Sample sizes for the experiments on PID 21, 56, and 84 were calculated using expected effect size and variance based on data of Iba-1-immunostained area (%) in the AP on PID 35. The Kolmogorov–Smirnov test was used as a test of normality. Unpaired *t*-test (two-sided) was used for comparison between two groups. When the normal distribution was not confirmed, the Mann–Whitney *U* test was used to compare the mean ranks of two groups. Three groups were compared by one-way analysis of variance (ANOVA) followed by a Bonferroni post hoc test. Correlation analysis was performed using Spearman’s rank correlation. To classify cells according to morphological parameters, principal component analysis (PCA) and hierarchical clustering analysis (HCA) were used. Frequencies of categorical variables were compared using the chi-square test. Differences were considered significant when the *p* value was < 0.05.

## Results

### Time course of microglial changes in the AP during CIA progression

Our preliminary observations indicated that the shape of cerebral ventricles of the DBA/1J mice we used for to create the CIA model were not identical to more popularly used mouse strains. Because information about the brain morphology of DBA/1J mice is limited, we first identified the major three sCVOs by observing extravascular leakage of FITC (Supplementary Figure 3), which is the most straightforward definition of the sCVO—where BBB function is attenuated [19, 26]. Although the localization and form of SFO was observably different from that of other strains, such as C57BL (Supplementary Figure 3B), the shape and localization of the AP was almost identical. Therefore, we performed the following analyses using the conventional location of the AP: immediately beneath the fourth ventricle.

**Fig. 1:**
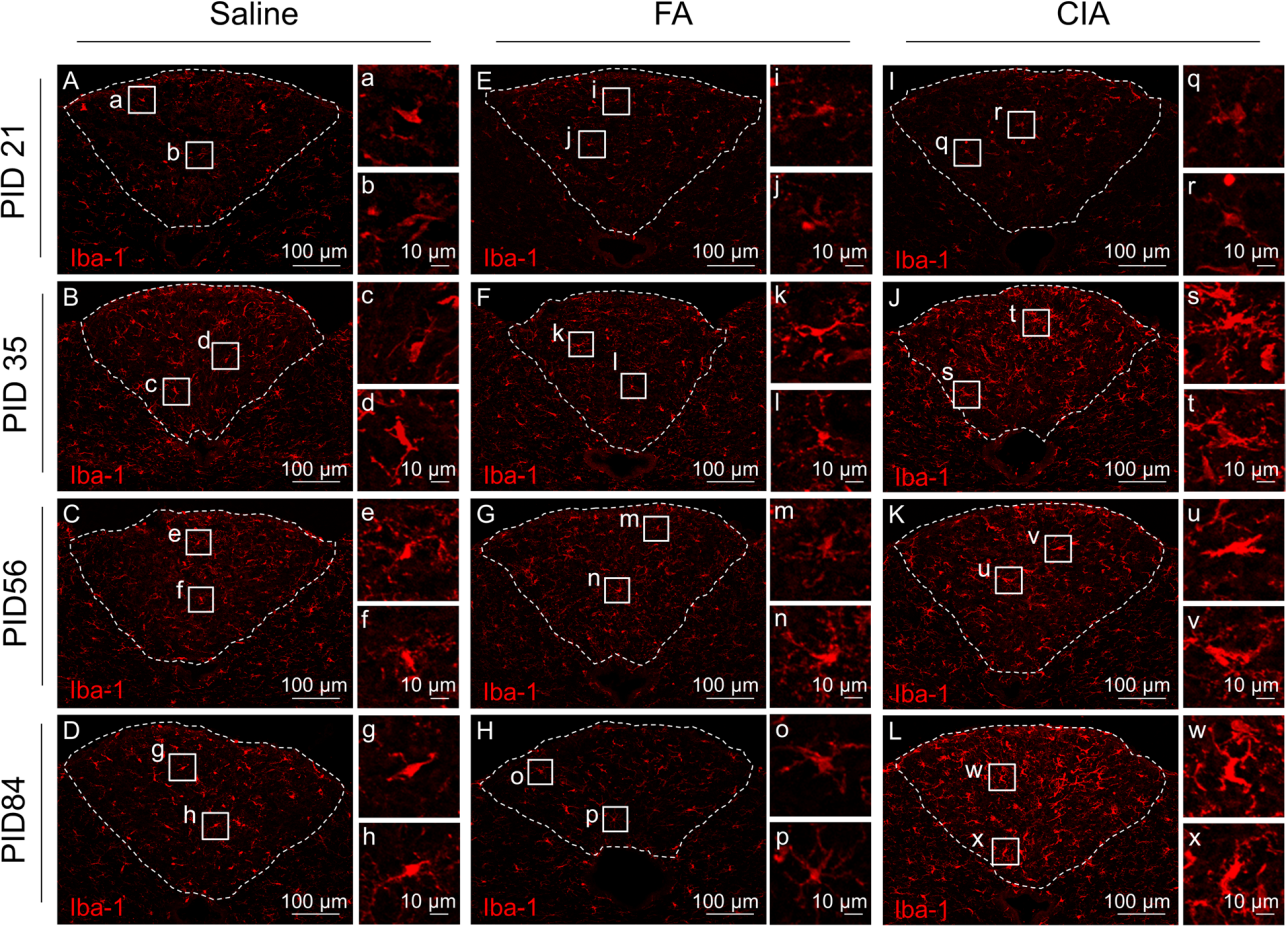
Microglia in the AP during the progression of CIA. **A–L** Representative images of immunostaining for Iba-1 in the AP during each disease phase in saline (**A–B**), FA (**E–H**), and CIA groups (**I–L**). Dashed lines indicate region of the AP. Boxed areas are shown at higher magnification on the right (**a–x**). Abbreviations: AP, area postrema; CIA, collagen-induced arthritis; FA, Freund’s adjuvant; Iba-1, ionized calcium-binding protein-1

**Fig. 2:**
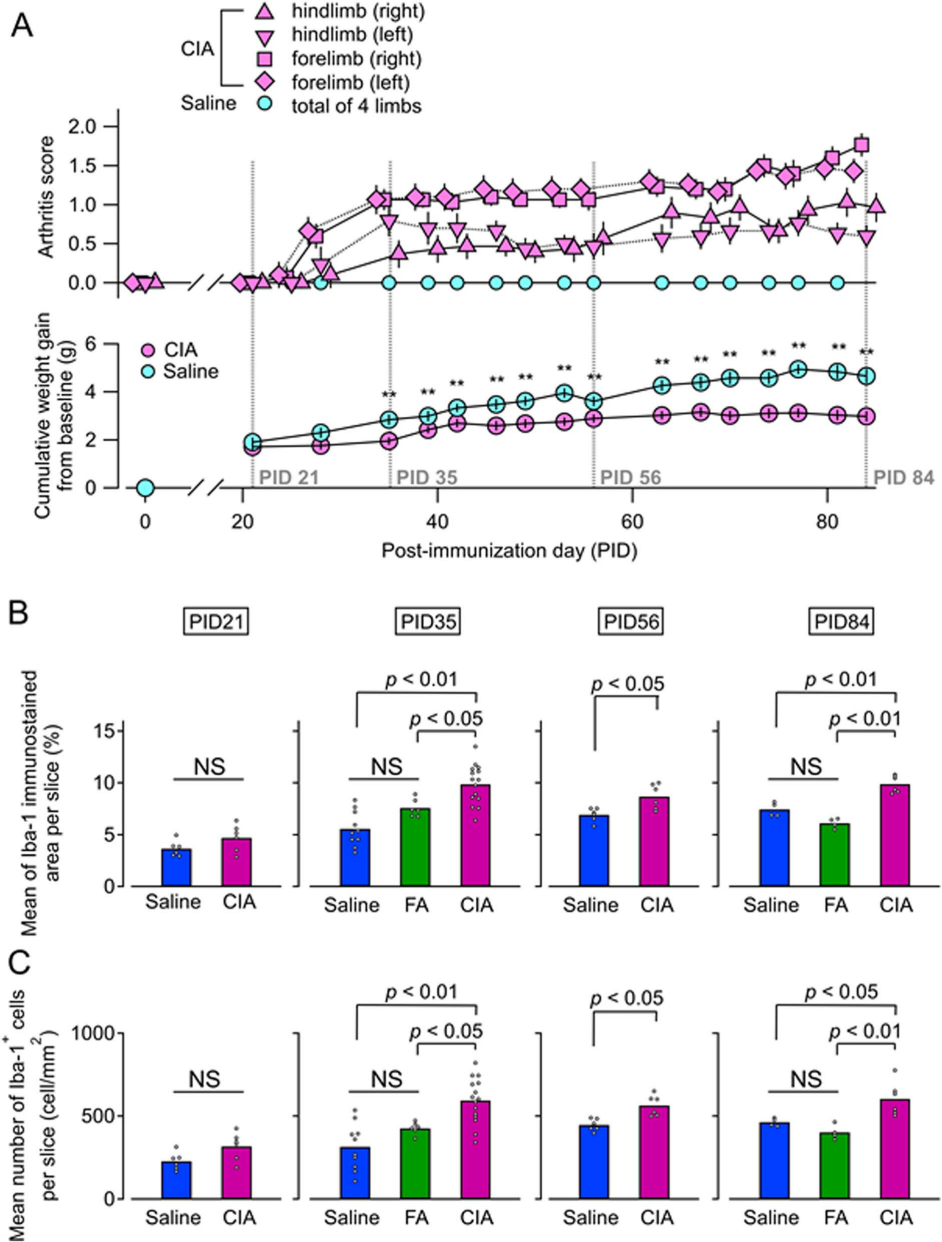
Arthritis scores, body weight changes, and Iba-1 immunoreactivity by disease phase. **A** Time course of arthritis scores (upper panel) and body weight changes (lower panel) (CIA, *n* = 30; saline, *n* = 24). Dashed line represents the day of brain analysis. No arthritic mice were found on PID 21. Arthritis scores gradually increased after onset. Joint swelling was sustained on PID 84. Cumulative weight gain from baseline (PID 0) in the CIA group were significantly lower compared with the saline group after PID 35 (**p < 0.01, by unpaired *t*-test). **B** Quantitative analysis of ionized Iba-1-immunostained area (%). **C** Quantitative analysis of numbers of Iba-1-positive cells. There were no significant differences between groups on PID 21 by unpaired *t*-test (CIA, *n* = 6; saline, *n* = 6). Conversely, area and number were significantly larger in the CIA group compared with the saline group on PID 35 (CIA, *n* = 15; FA, *n* = 6; saline, *n* = 10), PID 56 (CIA, *n* = 6; saline, *n* = 6), and PID 84 (CIA, *n* = 6; FA, *n* = 4; saline, *n* = 4) by unpaired *t*-test. Abbreviation: CIA, collagen-induced arthritis; FA, Freund’s adjuvant; Iba-1, ionized calcium-binding adaptor protein-1; NS, non-significant; PID, post-immunization day

Immunostaining for Iba-1 in the AP was performed during pre-onset (PID 21), establishment (PID 35), and chronic (PID 56 and 84) phases in saline, FA, and CIA mice (Fig. 1A). As negative controls, immunostaining using a nonspecific rabbit IgG antibody were also performed (Supplementary Figure 4). To determine the influence of arthritis, saline and FA groups served as controls for the CIA group. More extensive Iba-1-immunoreactivity was observed in the CIA group compared with saline and FA groups at PID 35, 56, and 84 (Fig. 1I, J, K, and L). At these disease phases, Iba-1-immunoreactivity in the CIA group was manifest in swollen cell bodies (Fig. 1q–x). Compared with the saline group, the FA group exhibited comparable Iba-1 immunoreactivity at all phases (Fig. 1E–H). Iba-1-immunoreactivity in saline and FA groups mainly emanated from small cell bodies (Fig. 1a–h and i–p). This sustained, specific pattern of microglial activation was associated with symptoms that developed during the course of arthritis, including joint swelling (arthritis scores) and body weight changes. Joint swelling persisted until PID 56 and PID 84 (Fig. 2A). Body weight changes in the CIA group were significantly lower compared with the saline group at these phases (Fig. 2A). Likewise, mRNA expression of IL-1β and IL-6 was significantly higher in the joints of all four limbs in the CIA group compared with the saline group on PID 56 and PID 84 (Supplementary Figure 5). These findings demonstrate that inflammatory symptoms and biochemical activation persist until the chronic phase, in which microglial activation remained. To quantitatively confirm this association, we performed quantitative analyses of microglia number and density on PID 21, 35, 56, and 84 (Fig. 2B, C). Iba-1-immunostained area (%) (Fig. 2B) and numbers of microglia/mm^2^ (Fig. 2C) were significantly higher in the CIA group compared with the saline group at PID 35, 56, and 84. Neither parameter differed significantly on PID 21, similar to arthritis symptoms. On PID 35 and PID 84, quantitative analysis of the FA group was also performed. Compared with the FA groups, the Iba-immunostained area (%) and number of microglia/mm^2^ were significantly higher in the CIA group on both PID 35 and PID 84. There were no significant differences between saline and FA groups (Fig. 2B, C). Therefore, only the saline group was used as a control group for the remainder of AP experiments. We also similarly examined Iba-1 immunostaining in the SFO and OVLT (Supplementary Figure 6A and C) on PID 35. There were no significant differences between CIA and FA groups in Iba-1-immunostained area (%) or number of microglia (/mm^2^) in these regions (Supplementary Figure 6B and D).

### Microglial morphology in the AP during various disease phases

**Fig. 3:**
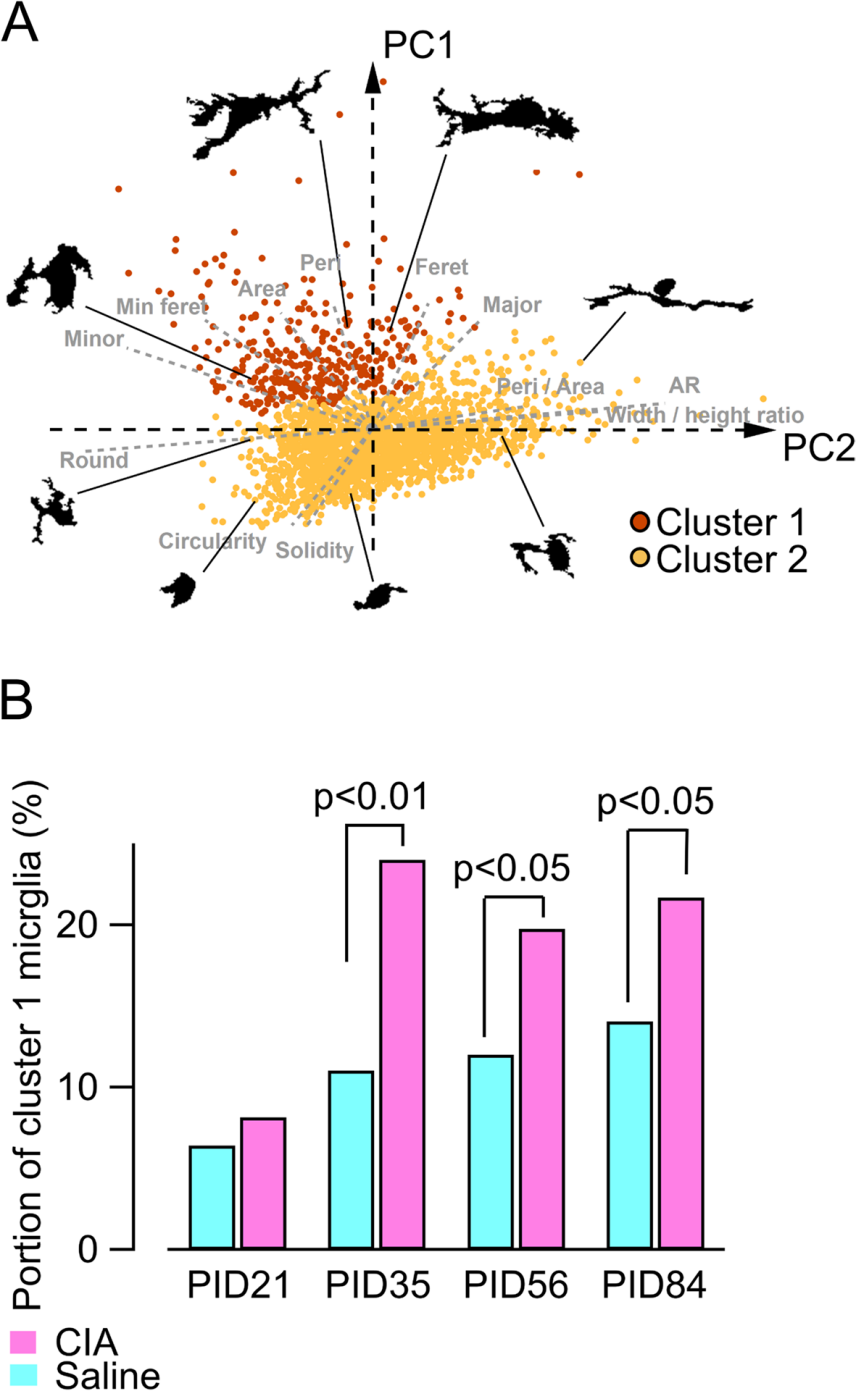
Morphological classification of microglia and increase of activated microglia during arthritis. **A** Morphological plots of each cell (2118 microglia cells from 51 mice) on the first two principal component (PC-1 and PC-2) coordinate planes with examples of their morphology (PID 21: CIA, *n* = 6, saline, *n* = 6; PID 35: CIA, *n* = 7, saline, *n* = 10; PID 56: CIA = 6, saline, *n* = 6; and PID 84: CIA, *n* = 6, saline, *n* = 4). Microglia were classified into cluster-1 and cluster-2 by hierarchical clustering analysis. Gray dashed lines reflect loading plots. **B** Proportion of cluster-1 microglia during each disease phase. There were no differences between groups on PID 21 by chi-squared test. Conversely, the proportion in the collagen-induced arthritis (CIA) group was higher than the saline groups on PID 35, 56, and 84 by chi-squared test. Abbreviations: AR, aspect ratio; Feret, Feret diameter; Peri, perimeter; Peri/Area, ratio of perimeter to area; PID, post-immunization day; Min Feret, minimum Feret diameter; Minor, minor diameter; Major, major diameter; Round, roundness

To compare microglial morphology in the CIA and saline groups, morphological parameters of Iba-1 immunostaining in the AP were measured on PID 21, 35, 56, and 84 (Supplementary Figure 2). On PID 21, no parameter was significantly different between groups (Supplementary Figure 7). However, several parameters differed significantly between groups on PID 35, 56, and 84 (Supplementary Figures 8, 9 and 10). In particular, a larger perimeter and smaller circularity were both significant in the CIA group on PID 35, 56, and 84. All morphological parameters of 2118 cells from 51 mice on PID 21, 35, 56, and 84 (CIA, *n* = 25; saline, *n* = 26) were subjected to PCA to identify principal components. The first principal component (PC-1) and second principal component (PC-2) accounted for 74.4% of the observed variability (Supplementary Table 1). To classify microglia according to the measured morphological parameters, HCA was performed using PC-1 and PC-2, which automatically divided all microglia into two clusters (Fig. 3A). Cluster-1 microglia were characterized by swollen cell bodies, which is one of the characteristics of activated microglia [27], and identified by higher perimeter, area, and minor diameter values, and a lower circularity value. Conversely, cluster-2 microglia were characterized by small cell bodies. The proportion of cluster-1 microglia in the CIA group was significantly larger than the saline group on PID 35, 56, and 84, but not PID 21 (Fig. 3B).

### Correlation between number of cluster-1 microglia and body weight changes

**Fig. 4:**
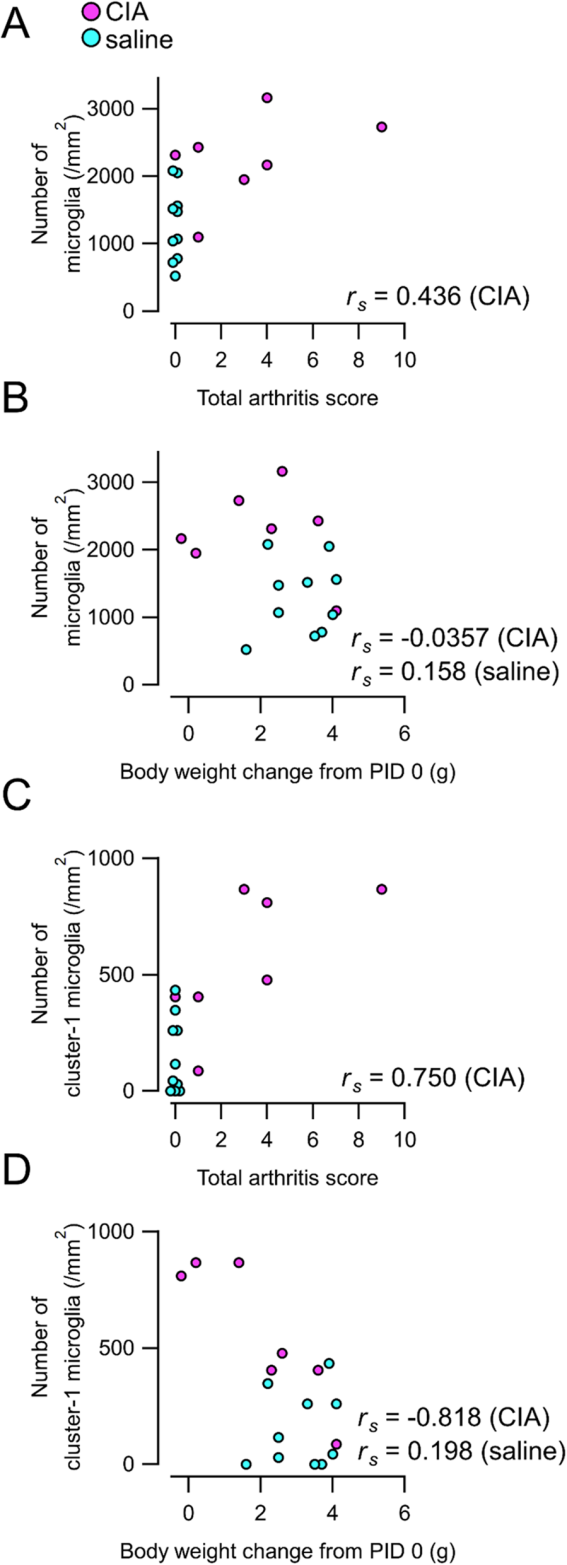
Correlation of microglia number with total arthritis scores and body weight changes. **A, B** Correlation of total microglia number with total arthritis scores (**A**) and body weight changes from PID 0 (**B**). **C, D** Correlation of number of cluster-1 microglia with total arthritis scores (**C**) and body weight changes (**D**). The number of cluster-1 microglia significantly correlated with body weight changes (*r*_*s*_ = − 0.818, *n* = 7, *p* = 0.0244; Spearman rank correlation coefficient). Abbreviations: CIA, collagen-induced arthritis; PID, post-immunization day

We examined correlations between microglia number and total arthritis score and body weight changes on PID 35 (Fig. 4). The number of microglia showed no correlation with total arthritis score or body weight changes from PID 0 (Fig. 4A, B). However, examining within each cluster, we found that the number of cluster-1 microglia significantly correlated with body weight changes from PID 0 (Fig. 4D). Conversely, the number of cluster-2 microglia did not correlate with body weight changes (data not shown). There were no significant correlations between microglia number in each cluster and total arthritis score (Fig. 4C).

### Increase of IL-1β-positive microglia in the AP

**Fig. 5:**
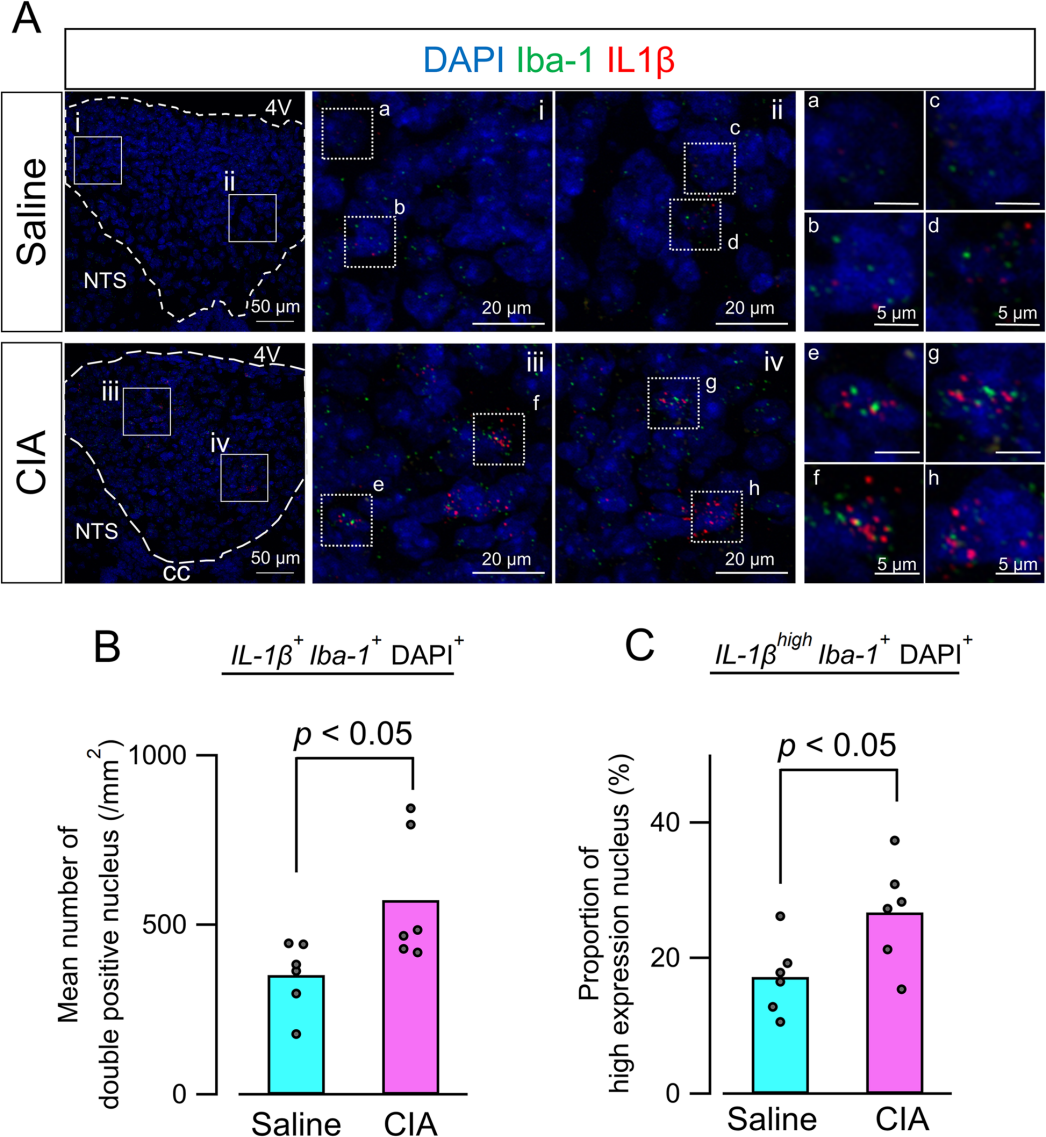
Microglial IL-1β mRNA expression in the AP. **A** Representative merged images of IL-1β (red) and Iba-1 (green) mRNA expression in the AP. Dashed lines indicate region of the AP (left panels). Magnified images of boxed areas in left panels are shown in middle panels (**i–iv**). High-magnification images of dashed boxed area in middle panels are shown in the right panels (**a–h**). In the CIA group, increased numbers of nuclei co-expressing IL-1β and Iba-1 were observed. Additionally, Iba-1-positive nuclei exhibiting high-density IL-1β mRNA expression were more frequently detected in the CIA group. **B** Quantitative analysis of the number of nuclei co-expressing IL-1β and Iba-1. Numbers of nuclei were significantly increased in the CIA group (*n* = 6) compared with the saline group (*n* = 6) by Mann–Whitney *U* test. **C** Quantitative analysis of proportions of Iba-1-positive nuclei with high-density IL-1β mRNA expression. Proportions were significantly increased in the CIA group (CIA, *n* = 6; saline, *n* = 6) by unpaired *t*-test. Abbreviations: AP, area postrema; CIA, collagen-induced arthritis; Iba-1, ionized calcium-binding adaptor protein-1; IL-1β interleukin-1β

IL-1β is one of the molecules produced by activated microglia [28]. To detect biochemical activation of microglia, we performed single-molecule RNA in situ hybridization and observed microglial IL-1β mRNA expression in the AP on PID 35. Images using control probes were shown in Supplementary Figure 11. By combining IL-1β and Iba-1 mRNA expression with DAPI staining, co-expression of IL-1β and Iba-1 mRNA was found in the AP (Fig. 5A). In the CIA group, there was an increase in co-expressing nuclei, and several nuclei exhibited high-density expression of IL-1β mRNA (Fig. 5A). Quantitative analysis revealed that the number of co-expressing nuclei (IL-1β^+^Iba-1^+^DAPI^+^) (i.e., IL-1β-positive microglia) was significantly increased in the CIA group (Fig. 5B). To quantify microglia with high-density IL-1β mRNA, the number of high-IL-1β expression nuclei with coexistence *Iba-1* puncta (IL-1β^high^Iba-1^+^DAPI^+^) were also counted. Consequently, the proportion of IL-1β^high^Iba-1^+^ nuclei to IL-1β^+^Iba-1^+^ nuclei significantly increased in the CIA group compared with the saline group (Fig. 5C).

### Correlation of IL-1β mRNA expression in the medulla containing the AP with sucrose-preference alterations in CIA model mice

**Fig. 6:**
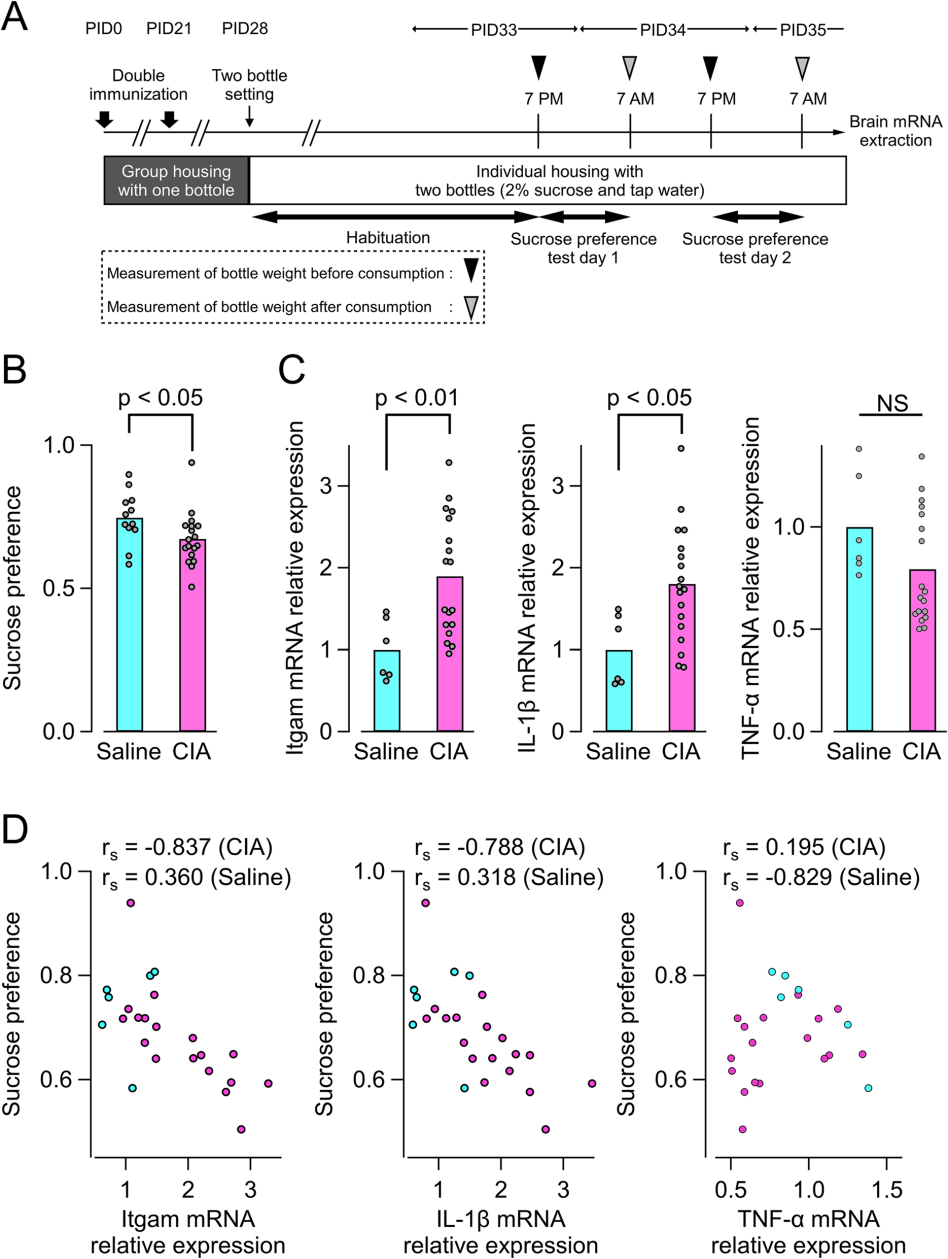
Correlation of IL-1β expression level in the AP of brain with depressive behavior of CIA mice. **A** Experimental scheme for behavioral test. **B** Results of sucrose preferences test (CIA, *n* = 18; saline, *n* = 12). The preference for sucrose in the CIA group was significantly lower compared with the saline group by unpaired *t*-test. **C** mRNA expression levels of a microglial activation marker (Itgam, left panel) and cytokines (IL-1β, middle panel; TNF-α, right panel) in medulla containing the AP (CIA, *n* = 18; saline, *n* = 6). Itgam and lL-1β were significantly higher in the CIA group. **D** Correlation of medullary mRNA expression with sucrose preferences (CIA, *n* = 18; saline, *n* = 6). Preference for sucrose in the CIA group significantly correlated with expression of Itgam (*r*_*s*_ = − 0.837, *n* = 18, *p* = 0.0000013; Spearman rank correlation coefficient) and IL-1β (*r*_*s*_ = − 0.788, *n* = 7, *p* = 0.000104; Spearman rank correlation coefficient), but not TNF-α (*r*_*s*_ = 0.195, *n* = 7, *p* = 0.438; Spearman rank correlation coefficient). There were no significant correlations between preference for sucrose in the saline group and any mRNAs evaluated. Abbreviations: AP, area postrema; CIA, collagen-induced arthritis; IL-1β, interleukin-1β; PID, post-immunization day; TNF-α, tumor necrosis factor-α

The AP is rich in small neurons that project to brain regions involved in various homeostatic functions, such as the nucleus of the solitary tract (NTS), dorsal motor nucleus of the vagus nerve, and the parabrachial nucleus [27–29]. Because all these areas play essential roles in regulation of appetite and feeding behaviors, we examined whether CIA mice show changes in nutrient-associated preferences in a manner correlated with cytokine expression in the AP (Fig. 6A). On PID 35, CIA mice exhibited a lowered preference for the sucrose water compared with saline-treated mice (Fig. 6B). mRNA expression levels of Itgam, a microglial activation marker, and IL-1β in medulla containing the AP were significantly increased in the CIA group (Fig. 6C). Moreover, mRNA expression of Itgam and IL-1β were negatively correlated with sucrose preferences in the CIA group, but not the saline-treated group (Fig. 6D). There were no significant correlations between sucrose preference and mRNA expression levels of TNF-α (Fig. 6D), IL-6, or transforming growth factor β (TGF-β) in the brainstem (Supplementary Figure 13).

## Discussion

RA is a chronic inflammatory disease resulting from persistent autoimmune activation in the articular tissue. It has been established that a sustained increase in plasma cytokines underlies arthritis symptoms during the clinical active period, and even after the remission of arthritis activity for patients with RA [29]. Accordingly, such prolonged activation of peripheral inflammation is thought to mediate the various extra-articular symptoms that persist throughout the duration of RA, such as the diverse expression of neuropsychological complications [6]. For example, the severity of depression in patients with RA (as evaluated with the Hospital Anxiety and Depression Scale) is significantly correlated with blood levels of IL-6 and IL-17 in patients with RA [30, 31]. Likewise, improvement of arthritis symptoms after anti-cytokine pharmacotherapy in patients with RA is often accompanied by partial improvement of depression [12]. This evidence suggests that sustained immune activation may well underlie the neuropsychological symptoms experienced by patients with RA and observed in experimental RA models [20, 22]. Such a possibility is also supported by findings in patients without RA; indeed, dysregulation of peripheral cytokines is associated with severe and chronic or treatment-resistant depressive disorders, as well as various neuropsychological symptoms in humans [32–34] and aberrant behaviors in animals [35].

The next question we asked was how immune activation in the periphery continues to affect brain neuronal activity during persistent RA. Here, we found that microglia in the AP of experimental RA model mice exhibited sustained activation characterized by changes in morphology and IL-1β expression during a long period (up to PID 84). The CIA model used reportedly shows joint symptoms up to 120 days after initial immune activation [36]. The present study is the first to demonstrate that sustained microglial activation in the AP accompanied persistent peripheral inflammation. Moreover, morphological activation of microglia was accompanied by a decrease in body weight and attenuated preference to sucrose. Therefore, our findings support the notion that AP microglia are one of the interfaces for transmitting peripheral inflammatory information to the CNS throughout the long duration of RA. The relevance of these findings and possible mechanism are discussed below.

### Long-lasting microglial activation in the AP

In this study, sustained microglial activation was observed specifically in the AP, one of the sCVOs by which peripheral inflammatory mediators can directly and rapidly affect brain cells [24]. However, as a site of direct humoral contact, the reported responses of AP cells to peripheral immune challenges mostly describe acute and early responses to short-lasting systemic inflammation. For example, intravenous injection of IL-1β and IL-6 led to expression of nuclear factor-kappa B [16], signal transducer activator of transcription 3 [17], and c-fos [37] in sCVOs at 30 min or 60 min post-injection. Consequently, such acute signals rapidly activate microglia, which are abundant in sCVOs, allowing them to act as first-line responders in the brain to peripheral inflammation in animal models. For example, orofacial CFA injection increased numbers of microglia in the AP of mice increases at 48 h post-injection [38], bleomycin-induced lung injury altered the size and branch length of AP microglia on the seventh day post-bleomycin [39], and dextran sulfate-induced colitis resulted in increased numbers and morphological changes of microglia in the AP of mice approximately 7–10 days post-establishment [40].

Despite evidence showing acute and early microglial responses in sCVOs, information regarding sCVO microglial activation during the later “chronic” phases of models with persistent inflammation remains limited. Our study is the first to demonstrate that microglia in the AP of an RA mouse model remain morphofunctionally activated for more than 80 days from the first immunization. This long-term activation was not the simple result of acute inflammation by CFA because injection alone did not cause AP microglial activation during either the establishment (PID 35) or chronic (PID 84) phases.

Because microglial activation is strongly associated with neuronal excitation, synaptic transmission, adaptive synaptic plasticity [41, 42], and related behavioral outcomes [43], it is expected that persistently activated microglia in the AP would function as a faithful interpreter of the peripheral inflammatory state to the brain, even in the absence of sustained peripheral pathology. Further analysis of the mechanism underlying sustained microglial activation would facilitate understanding of how various CNS-mediated symptoms inherent to RA, such as chronic pain and depression, occur throughout the active phase, and persist even after remission or with low disease activity [7]. The present findings have opened the door to these unsolved issues.

Subjective morphological analysis of microglia in the AP, described for the first time in this study, was challenging because there is no a priori information as to 1) how to describe microglial morphologies in the AP, where microglia are densely packed and a portion are constitutively activated, unlike other brain areas [44], and 2) how to follow long-term changes in microglia during several months, which was the main objective of this study. We overcame these issues by applying previously reported cluster and PCA analyses [45, 46] to AP microglia, which permitted subjective and quantitative analysis of microglial morphology for as up to 84 days. We found that, in addition to a sustained increase in number, drastic changes in the morphology of microglia occurred in CIA mice, but not saline-treated mice, during the establishment-to-chronic phases. We applied an almost identical analytical approach as reported by Fernandez-Arjona and colleagues, who evaluated microglial morphology in the cortex, striatum, hippocampus [45], and thalamus [46] using micrographs sampled with a conventional two-dimensional (2-D) confocal microscope. They concluded that biochemical properties, such as IL-1β expression, of microglia correlated well with their morphological classifications [46]. Although there are inherent limitations of using 2-D projected images to analyze the properties of 3-D structured microglia, the results of clustering with a large sample size (*n* = 2118 cells) yielded clear and subjective measurements that supported the rationale for this approach.

### Signaling mechanisms other than the humoral pathway

Distinct pathways reportedly underlie peripheral-to-CNS signaling under pathological conditions, such as direct humoral pathways at sCVOs, vagus afferent-mediated pathways, and sensory nerve/spinal-dorsal horn mediated pathways [8, 9]. The activation of AP microglia observed in this study likely resulted from direct humoral signaling from peripheral circulation to the AP through its weak BBB [44], as discussed above. The morphofunctional activation of microglia observed with long-lasting RA is reminiscent of the potentiated proliferation, increase in numbers of microglia with activated morphology, and increase in IL-1β mRNA expression detected after injections of LPS or CFA [38, 47], to which sCVOs are the primary and earliest responding structure [48]. Although involvement of a vagal afferent pathway in RA-associated microglial activation cannot be fully ruled out, this neural pathway may play only a minor role because microglial activation was highly limited to the AP and almost absent in the adjacent NTS, where primary afferent fibers arising from the viscera terminate. Moreover, the number of vagal primary afferent projections to the AP is much smaller compared with the NTS [49]. In support of this, LPS-induced increases in the number of cells expressing IL-1β in the AP were not affected by bilateral vagotomy [50], suggesting limited involvement of vagal afferents in transmitting peripheral inflammatory information to the AP. Altogether, the most plausible pathway linking peripheral inflammation to AP microglial activation is direct humoral signaling through fenestrated capillaries in the AP. The leakage of cytokines from these “windows” could directly activate the various cytokine receptors expressed by microglia [51], thus altering their morphofunctional properties at or near the site of direct contacts.

### Correlation between appetitive behavior with AP microglia activation

The AP functions as a humoral sensor for circulating substances that regulate food intake and nutrition, such as amylin, cholecystokinin [52], TNF-α [53], and TGF-β [54]. The AP and dorsal vagal complex (DVC) play essential roles in central regulation of physiological functions related to energy intake. For example, chemogenetic activation of GABAergic neurons in the DVC elevates blood glucose concentration [55], while chemogenetic activation of DVC astrocytes reduces food intake and decreases body weight [56]. The present finding that sucrose preference was attenuated in a manner negatively correlated with brainstem mRNA expression levels of Itgam and IL-1β in CIA mice on PID 35 supports the notion that altered neuronal activities in the DVC following activation of AP microglia played a key role in CIA-dependent behavioral alterations. Although we did not perform the sucrose preference test during all CIA phases, the finding that a significant reduction in bodyweight lasting up to PID 84 correlated with the number of cluster-1 microglia favors the view that sustained peripheral inflammation enacts its consequences through AP microglia. A plausible interpretation is that activated microglia directly affected surrounding neuronal activities in the DVC to modify appetite for the higher-calorie drink, consistent with frequent reports of appetite loss in patients with RA [57, 58]. This possibility should be examined using techniques allowing selective inactivation or removal of microglia in the AP without the risk of globally affecting microglia or even macrophages; at the moment, this is a challenging but important future project [59, 60]. In the same CIA mouse model, Oto et al. reported a dissociation of behavioral complications (sleep disturbance and environment temperature preference) from the severity of arthritis after the improvement of arthritis by tofacitinib [20]. Therefore, examining whether the sustained alterations of AP microglia observed in the CIA model are reversible after treatment by disease-modifying antirheumatic drugs is intriguing.

## Conclusion

We demonstrated a markedly prolonged increase in numbers of microglia in the AP, which was accompanied by their morphological activation during persistent arthritis, more than 80 days from first immunization, in a CIA mouse model. Because RA is characterized by long-lasting immunoactivation, this finding advances our knowledge on how peripherally sustained inflammation continues to affect CNS function. Our findings raise the possibility that microglia in the AP are a durable messenger for this sustained influence. However, an important question still remains—how were AP microglia numbers increased? Because Iba-1 was used to identify microglia, possibilities may include proliferation of AP-resident microglia by exuded cytokines, migration from surrounding brain structures in response to attraction by exuded cytokines, and invasion of peripheral monocytes [47]. Identification of the precise mechanism of long-lasting influence will facilitate development of appropriate therapeutic strategies to perturb neuropsychological complications in patients with RA.

## Supplementary Information

**Additional file 1: Supplementary Table 1.** Component loading and variances of principal component analysis. PC-1, first principal component; PC-2, secondary principal component. **Supplementary Figure 1.** Four regions of interest (ROIs) for morphological analysis. A: Representative image showing the location of ROIs (124 μm × 93 μm, yellow and white boxes). ROIs were placed on the four main divisions described in previous reports [23, 24]. A blinded examiner placed ROIs by referring to immunostaining of glial fibrillary acidic protein (GFAP). B: ROIs on the image of immunostaining of ionized calcium-binding adaptor protein-1 (Iba-1). C: Higher magnification image of yellow boxed area in A and B. D: Binary image of C. Iba-1-staining was transformed to binary images using the “triangle methods”. **Supplementary Figure 2.** Representative examples of twelve measured morphological parameters. Binary images of ionized calcium-binding adaptor protein-1 (Iba-1) staining, like Supplementary Figure 1D, were used for the analysis. Area (μm^2^), perimeter length (μm), and circularity were measured using the outer edge (indicated by the red line). Major diameter, minimum diameter, aspect ratio, and roundness were measured using the best fitting ellipse (indicated by the blue line). The purple line shows the Feret diameter. Solidity was calculated using the convex hull (indicated by the green line). Width and height were measured using the bounding rectangle (indicated by the orange line). PID, post-immunization day. **Supplementary Figure 3.** Detection of sensory circumventricular organs (sCVOs) in DBA/1J mice. A: Illustration showing the general location of three sCVOs (indicated in green) in mouse brain. B: Upper panels show extravascular leakage of fluorescein isothiocyanate (FITC) in sCVOs of naïve DBA/1J mouse. After transcardial perfusion of FITC, fluorescence was diffusely observed in three regions adjacent to the ventricles. The lower panels show CD31 immunoreactivity in sCVOs. Immunostaining for CD31, a marker of endothelial cells, showed high vascular density in FITC-leakage areas (dashed lines). These findings conformed to characteristics of sCVOs described in previous reports [35, 37]. Thus, these areas were identified as sCVOs. 3V, third ventricle; 4V, fourth ventricle; AP, area postrema; OVLT, organum vasculosum of the lamina terminalis; sCVOs, sensory circumventricular organs; SFO, subfornical organ. **Supplementary Figure 4.** Representative images of immunostaining for ionized calcium-binding protein-1 (Iba-1; left panels) and rabbit isotype IgG (negative control; right panels) in the FA (4A) and CIA (4B) group on PID 35. In both groups, pairs of immunostainings images for Iba-1 and isotype controls were obtained from sets of consecutive 20 μm sections. 4V, fourth ventricle; NTS, nucleus of solitary tract; cc, central canal. **Supplementary Figure 5.** mRNA expression of *IL-1β* and *IL-6* in the joints of all four limbs of saline and collagen-induced arthritis mice on PID 56 (upper panel) and 84 (lower panel) by quantitative real-time PCR experiments. The values were normalized to the average of the saline group. Each circle represents value of a single mouse. *IL-1β* and *IL-6* mRNA expression in the CIA group were significantly higher compared with the saline group on both PID 56 (Saline vs. CIA: *IL-1β*, 1.00 ± 0.152 [*n* = 6] vs. 7.79 ± 1.11 [*n* = 12], *p* < 0.001; *IL-6*, 1.00 ± 0.255 [*n* = 6] vs. 49.3 ± 13.0 [*n* = 12], *p* = 0.0196; Student’s *t*-test) and PID 84 (Saline vs. CIA: *IL-1β*, 1.00 ± 0.13 [*n* = 9] vs. 5.36 ± 0.987 [*n* = 22], *p* = 0.0066; *IL-6*, 1.00 ± 0.201 [*n* = 9] vs. 13.0 ± 3.12 [*n* = 22], *p* = 0.0152; Student’s *t*-test). CIA, collagen-induced arthritis; *IL-1β*, interleukin 1 beta; *IL-6*, interleukin 6; PID, post-immunization day. **Supplementary Figure 6.** Microglia in the subfornical organs and organum vasculosum laminae terminalis (OVLT). A: Representative images of Iba-1 immunostaining in the subfornical organs (SFO). B: Quantitative analysis in the SFO. There were no significant differences between groups by unpaired t-test (CIA, n = 8; FA, *n* = 5). C: Representative images of Iba-1 immunostaining in the organum vasculosum laminae terminalis (OVLT). D: Quantitative analysis in the OVLT. There were no significant differences between groups by unpaired t-test (CIA, *n* = 8; FA, *n* = 5). Abbreviations: 4V: fourth ventricle; NTS, nucleus of the solitary tract; cc central canal; NS, non-significant. **Supplementary Figure 7.** Twelve morphological parameters of Iba-1-positive cells on PID 21. Each dot (blue: saline, magenta: CIA) represents morphological value of a single cell. There were no differences in any parameter on PID 21. (Saline vs. CIA: area, 53.9 ± 2.81 μm^2^ vs. 56.1 ± 2.42 μm^2^, *p* = 0.813; perimeter, 68.0 ± 3.64 μm vs. 69.7 ± 2.86 μm, *p* = 0.826; ratio of perimeter to area, 0.397 ± 0.011 vs. 0.40 ± 0.009, *p* = 0.827; ratio of width to height, 1.57 ± 0.066 vs. 1.57 ± 0.051, *p* = 0.851; major diameter, 12.4 ± 0.45 μm vs. 12.6 ± 0.298 μm, *p* = 0.504; minor diameter, 5.54 ± 0.163 μm vs. 5.64 ± 0.159 μm, *p* = 0.674; circularity, 0.191 ± 0.013 vs. 0.188 ± 0.009, *p* = 0.925; Feret diameter, 17.6 ± 0.714 μm vs. 17.9 ± 0.534 μm, *p* = 0.742; minimum Feret diameter, 8.96 ± 0.347 μm vs. 8.92 ± 0.281 μm, *p* = 0.582; aspect ratio, 2.46 ± 0.15 vs. 2.46 ± 0.094, *p* = 0.844; roundness, 0.488 ± 0.022 vs. 0.479 ± 0.015, *p* = 0.845; solidity, 0.517 ± 0.016 vs. 0.536 ± 0.012, *p* = 0.325; Mann–Whitney *U* test). Iba-1, ionized calcium-binding adaptor protein-1; PID, post-immunization day. **Supplementary Figure 8.** Twelve morphological parameters of Iba-1-positive cells on PID 35. Each dot (blue: saline, magenta: CIA) represents morphological value of a single cell. Nine parameters showed significant differences between groups (Saline vs. CIA: area, 59.6 ± 1.72 μm^2^ vs. 77.7 ± 3.0 μm^2^, *p* < 0.001; perimeter, 59.6 ± 1.72 μm vs. 101.5 ± 3.02 μm, *p* < 0.001; ratio of perimeter to area, 0.403 ± 0.006 vs. 0.444 ± 0.006, *p* < 0.001; ratio of width to height, 1.63 ± 0.033 vs. 1.71 ± 0.034, *p* = 0.175; major diameter, 13.2 ± 0.243 μm vs. 14.8 ± 0.303 μm, *p* < 0.001; minor diameter, 5.68 ± 0.092 μm vs. 6.24 ± 0.119 μm, *p* < 0.05; circularity, 0.192 ± 0.007 vs. 0.133 ± 0.005, *p* < 0.001; Feret diameter, 19.4 ± 0.423 μm vs. 22.6 ± 0.484 μm, *p* < 0.001; minimum Feret diameter, 9.23 ± 0.2 μm vs. 10.8 ± 0.236 μm, *p* < 0.001; aspect ratio, 2.58 ± 0.068 vs. 2.62 ± 0.06, *p* = 0.617; roundness, 0.476 ± 0.01 vs. 0.456 ± 0.008, *p* = 0.128; solidity, 0.513 ± 0.008 vs. 0.455 ± 0.006, *p* < 0.001; Mann–Whitney *U* test). Iba-1, ionized calcium-binding adaptor protein-1; PID, post-immunization day. **Supplementary Figure 9.** Twelve morphological parameters of Iba-1-positive cells on PID 56. Each dot (blue: saline, magenta: CIA) represents morphological value of a single cell. Five parameters showed significant differences between groups (Saline vs. CIA: area, 59.6 ± 2.2 μm^2^ vs. 70.1 ± 2.58 μm^2^, *p* < 0.01; perimeter, 75.0 ± 2.78 μm vs. 87.7 ± 2.95 μm, *p* < 0.01; ratio of perimeter to area, 0.406 ± 0.084 vs. 0.407 ± 0.007, *p* = 0.987; ratio of width to height, 1.65 ± 0.046 vs. 1.59 ± 0.031, *p* = 0.475; major diameter, 13.3 ± 0.279 μm vs. 14.1 ± 0.273 μm, *p* = 0.105; minor diameter, 5.61 ± 0.130 μm vs. 6.07 ± 0.116 μm, *p* < 0.01; circularity, 0.188 ± 0.009 vs. 0.164 ± 0.007, *p* < 0.05; Feret diameter, 19.5 ± 0.516 μm vs. 20.9 ± 0.498 μm, *p* = 0.0953; minimum Feret diameter, 9.18 ± 0.271 μm vs. 10.3 ± 0.249 μm, *p* < 0.001; aspect ratio, 2.59 ± 0.078 vs. 2.52 ± 0.059, *p* = 0.530; roundness, 0.453 ± 0.012 vs. 0.462 ± 0.01, *p* = 0.532; solidity, 0.51 ± 0.011 vs. 0.488 ± 0.008, *p* = 0.116; Mann–Whitney *U* test). Iba-1, ionized calcium-binding adaptor protein-1; PID, post-immunization day. **Supplementary Figure 10.** Twelve morphological parameters of Iba-1-positive cells on PID 84. Each dot (blue: saline, magenta: CIA) represents morphological value of a single cell. Seven parameters showed significant differences between groups (Saline vs. CIA: area, 66.2 ± 2.53 μm^2^ vs. 76.7 ± 2.99 μm^2^, *p* = 0.201; perimeter, 76.3 ± 3.02 μm vs. 95.6 ± 3.61 μm, *p* < 0.001; ratio of perimeter to area, 0.37 ± 0.008 vs. 0.412 ± 0.006, *p* < 0.001; ratio of width to height, 1.56 ± 0.039 vs. 1.72 ± 0.04, *p* < 0.05; major diameter, 13.9 ± 0.347 μm vs. 15.1 ± 0.315 μm, *p* < 0.05; minor diameter, 5.97 ± 0.139 μm vs. 6.22 ± 0.14 μm, *p* = 0.907; circularity, 0.198 ± 0.01 vs. 0.146 ± 0.005, *p* < 0.001; Feret diameter, 19.5 ± 0.541 μm vs. 22.4 ± 0.559 μm, *p* < 0.001; minimum Feret diameter, 9.57 ± 0.283 μm vs. 10.5 ± 0.284 μm, *p* = 0.086; aspect ratio, 2.53 ± 0.09 vs. 2.76 ± 0.079, *p* = 0.216; roundness, 0.467 ± 0.013 vs. 0.448 ± 0.01, *p* = 0.217; solidity, 0.53 ± 0.011 vs. 0.475 ± 0.008, *p* < 0.001; Mann–Whitney *U* test). Iba-1, ionized calcium-binding adaptor protein-1; PID, post-immunization day. **Supplementary Figure 11.** Dendrogram by hierarchical clustering analysis using the first two principal components (PC-1 and PC-2). Microglia were classified into cluster 1 and cluster 2. **Supplementary Figure 12.** Representative images of RNAcope® for target mRNA probes (upper panels), positive control probes (middle panels), negative control probes (lower panels) in the AP. Dashed lines indicate region of the AP (left panels). Boxed areas (i-ix) are shown at higher magnification on right panels. **Supplementary Figure 13.** Correlation of interleukin-6 (IL-6) and transforming growth factor beta (TGF-β) mRNA expression in the AP level brain with sucrose preference of CIA mice. A and C: Relative expression of IL-6 (A) and TGF-β (C) mRNA in the AP level brain. In the CIA group, IL-6 mRNA expression was significantly larger by unpaired t-test (CIA, 1.85 ± 0.213, *n* = 18; saline 1.00 ± 0.281, *n* = 6). In TGF-β expression, there is no significantly difference between the CIA and saline groups by unpaired t-test (CIA, 0.79 ± 0.064, *n* = 18; saline 1.00 ± 0.104, *n* = 6) B and D: The correlation of brain IL-6 (B) and TGF-β (D) mRNA expression with the sucrose preferences. Significant correlations were not observed.

## Data Availability

The datasets used and/or analyzed during the current study are available from the corresponding author on reasonable request.

## Abbreviations

ANOVA: Analysis of variance
AP: Area postrema
BBB: Blood–brain barrier
CFA: Complete Freund’s adjuvant
CIA: Collagen-induced arthritis
CNS: Central nervous system
DAPI: 4′,6-Diamidino-2-phenylindole
FA: Freund’s adjuvant
FITC: Fluorescein isothiocyanate
GFAP: Glial fibrillary acidic protein
HCA: Hierarchical clustering analysis
Iba-1: Ionized calcium-binding adaptor molecule-1
IFA: Incomplete Freund’s adjuvant
IFN-γ: Interferon-gamma
IL: Interleukin
LPS: Lipopolysaccharide
OVLT: Organum vasculosum laminae terminalis
PB: Phosphate buffer
PBS: Phosphate-buffered saline
PC-1: First principal component
PC-2: Second principal component
PCA: Principal component analysis
PCR: Polymerase chain reaction
PFA: Paraformaldehyde
PID: Post-immunization days
RA: Rheumatoid arthritis
sCVOs: Sensory circumventricular organs
SFO: Subfornical organs
TNF: Tumor necrosis factor
