# Supplementary material for: Sustained microglial activation in the area postrema of collagen-induced arthritis mice
Source: Arthritis Res Ther. 2021 Oct 29;23:273. doi: 10.1186/s13075-021-02657-x (PMC8556992; doi:10.1186/s13075-021-02657-x)
Supplement: Supplementary file 1 — Additional file 1: Supplementary Table 1. Component loading and variances of principal component analysis. PC-1, first principal component; PC-2, secondary principal component. Supplementary Figure 1. Four regions of interest (ROIs) for morphological analysis. A: Representative image showing the location of ROIs (124 μm × 93 μm, yellow and white boxes). ROIs were placed on the four main divisions described in previous reports [23, 24]. A blinded examiner placed ROIs by referring to immunostaining of glial fibrillary acidic protein (GFAP). B: ROIs on the image of immunostaining of ionized calcium-binding adaptor protein-1 (Iba-1). C: Higher magnification image of yellow boxed area in A and B. D: Binary image of C. Iba-1-staining was transformed to binary images using the “triangle methods”. Supplementary Figure 2. Representative examples of twelve measured morphological parameters. Binary images of ionized calcium-binding adaptor protein-1 (Iba-1) staining, like Supplementary Figure 1D, were used for the analysis. Area (μm2), perimeter length (μm), and circularity were measured using the outer edge (indicated by the red line). Major diameter, minimum diameter, aspect ratio, and roundness were measured using the best fitting ellipse (indicated by the blue line). The purple line shows the Feret diameter. Solidity was calculated using the convex hull (indicated by the green line). Width and height were measured using the bounding rectangle (indicated by the orange line). PID, post-immunization day. Supplementary Figure 3. Detection of sensory circumventricular organs (sCVOs) in DBA/1J mice. A: Illustration showing the general location of three sCVOs (indicated in green) in mouse brain. B: Upper panels show extravascular leakage of fluorescein isothiocyanate (FITC) in sCVOs of naïve DBA/1J mouse. After transcardial perfusion of FITC, fluorescence was diffusely observed in three regions adjacent to the ventricles. The lower panels show CD31 immunoreactivity in sCVOs. Immu [file 13075_2021_2657_MOESM1_ESM.zip › Supplementary figure legends.docx]

Supplementary Table 1. Component loading and variances of principal component analysis.

|  | PC-1 | PC-2 |
| --- | --- | --- |
| Aspect ratio | 0.083 | 0.483 |
| Area | 0.374 | -0.151 |
| Circularity | -0.311 | -0.133 |
| Feret diameter | 0.405 | 0.092 |
| Major diameter | 0.347 | 0.176 |
| Minimum Feret diameter | 0.352 | -0.276 |
| Minor diameter | 0.261 | -0.405 |
| Perimeter/area | 0.072 | 0.249 |
| Perimeter | 0.413 | -0.066 |
| Roundness | -0.073 | -0.477 |
| Solidity | -0.308 | -0.107 |
| Width/height | 0.057 | 0.376 |
| Standard deviation | 2.33 | 1.87 |
| Proportion of variance | 0.454 | 0.29 |
| Cumulative proportion | 0.454 | 0.744 |

Abbreviations: first principal component, PC-1; secondary principal component, PC-2.

Supplementary Figure 1. Four regions of interest (ROIs) for morphological analysis. **A:** Representative image showing the location of ROIs (124 µm × 93 µm, yellow and white boxes). ROIs were placed on the four main divisions described in previous reports (23, 24). A blinded examiner placed ROIs by referring to immunostaining of glial fibrillary acidic protein (GFAP). **B:** ROIs on the image of immunostaining of ionized calcium-binding adaptor protein-1 (Iba-1). **C:** Higher magnification image of yellow boxed area in A and B. **D:** Binary image of **C**. Iba-1-staining was transformed to binary images using the “triangle methods”.

Supplementary Figure 2. Representative examples of twelve measured morphological parameters. Binary images of ionized calcium-binding adaptor protein-1 (Iba-1) staining, like Supplementary Figure 1D, were used for the analysis. Area (µm^2^), perimeter length (µm), and circularity were measured using the outer edge (indicated by the red line). Major diameter, minimum diameter, aspect ratio, and roundness were measured using the best fitting ellipse (indicated by the blue line). The purple line shows the Feret diameter. Solidity was calculated using the convex hull (indicated by the green line). Width and height were measured using the bounding rectangle (indicated by the orange line). PID, post-immunization day.

Supplementary Figure 3. Detection of sensory circumventricular organs (sCVOs) in DBA/1J mice. **A:** Illustration showing the general location of three sCVOs (indicated in green) in mouse brain. **B:** Upper panels show extravascular leakage of fluorescein isothiocyanate (FITC) in sCVOs of naïve DBA/1J mouse. After transcardial perfusion of FITC, fluorescence was diffusely observed in three regions adjacent to the ventricles. The lower panels show CD31 immunoreactivity in sCVOs. Immunostaining for CD31, a marker of endothelial cells, showed high vascular density in FITC-leakage areas (dashed lines). These findings conformed to characteristics of sCVOs described in previous reports (35, 37). Thus, these areas were identified as sCVOs. 3V, third ventricle; 4V, fourth ventricle; AP, area postrema; OVLT, organum vasculosum of the lamina terminalis; sCVOs, sensory circumventricular organs; SFO, subfornical organ.

Supplementary Figure 4. Representative images of immunostaining for ionized calcium-binding protein-1 (Iba-1; left panels) and rabbit isotype IgG (negative control; right panels) in the FA (**4A**) and CIA (**4B**) group on PID 35. In both groups, pairs of immunostainings images for Iba-1 and isotype controls were obtained from sets of consecutive 20 µm sections. 4V, fourth ventricle; NTS, nucleus of solitary tract; cc, central canal.

Supplementary Figure 5. mRNA expression of *IL-1β* and *IL-6* in the joints of all four limbs of saline and collagen-induced arthritis mice on PID 56 (upper panel) and 84 (lower panel) by quantitative real-time PCR experiments. The values were normalized to the average of the saline group. Each circle represents value of a single mouse. *IL-1β* and *IL-6* mRNA expression in the CIA group were significantly higher compared with the saline group on both PID 56 (Saline vs. CIA: *IL-1β*, 1.00 ± 0.152 [*n* = 6] vs. 7.79 ± 1.11 [*n* = 12], *p* < 0.001; *IL-6*, 1.00 ± 0.255 [*n* = 6] vs. 49.3 ± 13.0 [*n* = 12], *p* = 0.0196; Student’s *t*-test) and PID 84 (Saline vs. CIA: *IL-1β*, 1.00 ± 0.13 [*n* = 9] vs. 5.36 ± 0.987 [*n* = 22], *p* = 0.0066; *IL-6*, 1.00 ± 0.201 [*n* = 9] vs. 13.0 ± 3.12 [*n* = 22], *p* = 0.0152; Student’s *t*-test). CIA, collagen-induced arthritis; *IL-1β*, interleukin 1 beta; *IL-6*, interleukin 6; PID, post-immunization day.

Supplementary Figure 6. Microglia in the subfornical organs and organum vasculosum laminae terminalis (OVLT). **A:** Representative images of Iba-1 immunostaining in the subfornical organs (SFO). **B:** Quantitative analysis in the SFO. There were no significant differences between groups by unpaired t-test (CIA, n = 8; FA, n = 5). **C:** Representative images of Iba-1 immunostaining in the organum vasculosum laminae terminalis (OVLT). **D:** Quantitative analysis in the OVLT. There were no significant differences between groups by unpaired t-test (CIA, n = 8; FA, n = 5). Abbreviations: 4V: fourth ventricle; NTS, nucleus of the solitary tract; cc central canal; NS, non-significant.

Supplementary Figure 7. Twelve morphological parameters of Iba-1-positive cells on PID 21. Each dot (blue: saline, magenta: CIA) represents morphological value of a single cell. There were no differences in any parameter on PID 21. (Saline vs. CIA: area, 53.9 ± 2.81 µm^2^ vs. 56.1 ± 2.42 µm^2^, *p* = 0.813; perimeter, 68.0 ± 3.64 µm vs. 69.7 ± 2.86 µm, *p* = 0.826; ratio of perimeter to area, 0.397 ± 0.011 vs. 0.40 ± 0.009, *p* = 0.827; ratio of width to height, 1.57 ± 0.066 vs. 1.57 ± 0.051, *p* = 0.851; major diameter, 12.4 ± 0.45 µm vs. 12.6 ± 0.298 µm, *p* = 0.504; minor diameter, 5.54 ± 0.163 µm vs. 5.64 ± 0.159 µm, *p* = 0.674; circularity, 0.191 ± 0.013 vs. 0.188 ± 0.009, *p* = 0.925; Feret diameter, 17.6 ± 0.714 µm vs. 17.9 ± 0.534 µm, *p* = 0.742; minimum Feret diameter, 8.96 ± 0.347 µm vs. 8.92 ± 0.281 µm, *p* = 0.582; aspect ratio, 2.46 ± 0.15 vs. 2.46 ± 0.094, *p* = 0.844; roundness, 0.488 ± 0.022 vs. 0.479 ± 0.015, *p* = 0.845; solidity, 0.517 ± 0.016 vs. 0.536 ± 0.012, *p* = 0.325; Mann–Whitney *U* test). Iba-1, ionized calcium-binding adaptor protein-1; PID, post-immunization day.

Supplementary Figure 8. Twelve morphological parameters of Iba-1-positive cells on PID 35. Each dot (blue: saline, magenta: CIA) represents morphological value of a single cell. Nine parameters showed significant differences between groups (Saline vs. CIA: area, 59.6 ± 1.72 µm^2^ vs. 77.7 ± 3.0 µm^2^, *p* < 0.001; perimeter, 59.6 ± 1.72 µm vs. 101.5 ± 3.02 µm, *p* < 0.001; ratio of perimeter to area, 0.403 ± 0.006 vs. 0.444 ± 0.006, *p* < 0.001; ratio of width to height, 1.63 ± 0.033 vs. 1.71 ± 0.034, *p* = 0.175; major diameter, 13.2 ± 0.243 µm vs. 14.8 ± 0.303 µm, *p* < 0.001; minor diameter, 5.68 ± 0.092 µm vs. 6.24 ± 0.119 µm, *p* < 0.05; circularity, 0.192 ± 0.007 vs. 0.133 ± 0.005, *p* < 0.001; Feret diameter, 19.4 ± 0.423 µm vs. 22.6 ± 0.484 µm, *p* < 0.001; minimum Feret diameter, 9.23 ± 0.2 µm vs. 10.8 ± 0.236 µm, *p* < 0.001; aspect ratio, 2.58 ± 0.068 vs. 2.62 ± 0.06, *p* = 0.617; roundness, 0.476 ± 0.01 vs. 0.456 ± 0.008, *p* = 0.128; solidity, 0.513 ± 0.008 vs. 0.455 ± 0.006, *p* < 0.001; Mann–Whitney *U* test). Iba-1, ionized calcium-binding adaptor protein-1; PID, post-immunization day.

Supplementary Figure 9. Twelve morphological parameters of Iba-1-positive cells on PID 56. Each dot (blue: saline, magenta: CIA) represents morphological value of a single cell. Five parameters showed significant differences between groups (Saline vs. CIA: area, 59.6 ± 2.2 µm^2^ vs. 70.1 ± 2.58 µm^2^, *p* < 0.01; perimeter, 75.0 ± 2.78 µm vs. 87.7 ± 2.95 µm, *p* < 0.01; ratio of perimeter to area, 0.406 ± 0.084 vs. 0.407 ± 0.007, *p* = 0.987; ratio of width to height, 1.65 ± 0.046 vs. 1.59 ± 0.031, *p* = 0.475; major diameter, 13.3 ± 0.279 µm vs. 14.1 ± 0.273 µm, *p* = 0.105; minor diameter, 5.61 ± 0.130 µm vs. 6.07 ± 0.116 µm, p < 0.01; circularity, 0.188 ± 0.009 vs. 0.164 ± 0.007, *p* < 0.05; Feret diameter, 19.5 ± 0.516 µm vs. 20.9 ± 0.498 µm, *p* = 0.0953; minimum Feret diameter, 9.18 ± 0.271 µm vs. 10.3 ± 0.249 µm, *p* < 0.001; aspect ratio, 2.59 ± 0.078 vs. 2.52 ± 0.059, *p* = 0.530; roundness, 0.453 ± 0.012 vs. 0.462 ± 0.01, *p* = 0.532; solidity, 0.51 ± 0.011 vs. 0.488 ± 0.008, *p* = 0.116; Mann–Whitney *U* test). Iba-1, ionized calcium-binding adaptor protein-1; PID, post-immunization day.

Supplementary Figure 10. Twelve morphological parameters of Iba-1-positive cells on PID 84. Each dot (blue: saline, magenta: CIA) represents morphological value of a single cell. Seven parameters showed significant differences between groups (Saline vs. CIA: area, 66.2 ± 2.53 µm^2^ vs. 76.7 ± 2.99 µm^2^, *p* = 0.201; perimeter, 76.3 ± 3.02 µm vs. 95.6 ± 3.61 µm, *p* < 0.001; ratio of perimeter to area, 0.37 ± 0.008 vs. 0.412 ± 0.006, *p* < 0.001; ratio of width to height, 1.56 ± 0.039 vs. 1.72 ± 0.04, *p* < 0.05; major diameter, 13.9 ± 0.347 µm vs. 15.1 ± 0.315 µm, *p* < 0.05; minor diameter, 5.97 ± 0.139 µm vs. 6.22 ± 0.14 µm, *p* = 0.907; circularity, 0.198 ± 0.01 vs. 0.146 ± 0.005, *p* < 0.001; Feret diameter, 19.5 ± 0.541 µm vs. 22.4 ± 0.559 µm, *p* < 0.001; minimum Feret diameter, 9.57 ± 0.283 µm vs. 10.5 ± 0.284 µm, *p* = 0.086; aspect ratio, 2.53 ± 0.09 vs. 2.76 ± 0.079, *p* = 0.216; roundness, 0.467 ± 0.013 vs. 0.448 ± 0.01, *p* = 0.217; solidity, 0.53 ± 0.011 vs. 0.475 ± 0.008, *p* < 0.001; Mann–Whitney *U* test). Iba-1, ionized calcium-binding adaptor protein-1; PID, post-immunization day.

Supplementary Figure 11. Dendrogram by hierarchical clustering analysis using the first two principal components (PC-1 and PC-2). Microglia were classified into cluster 1 and cluster 2.

Supplementary Figure 12. Representative images of RNAcope® for target mRNA probes (upper panels), positive control probes (middle panels), negative control probes (lower panels) in the AP. Dashed lines indicate region of the AP (left panels). Boxed areas (i-ix) are shown at higher magnification on right panels.

Supplementary Figure 13. Correlation of interleukin-6 (IL-6) and transforming growth factor beta (TGF-β) mRNA expression in the AP level brain with sucrose preference of CIA mice. **A and C**: Relative expression of IL-6 (**A**) and TGF-β (**C**) mRNA in the AP level brain. In the CIA group, IL-6 mRNA expression was significantly larger by unpaired t-test (CIA, 1.85 ± 0.213, n = 18; saline 1.00 ± 0.281, n = 6). In TGF-β expression, there is no significantly difference between the CIA and saline groups by unpaired t-test (CIA, 0.79 ± 0.064, n = 18; saline 1.00 ± 0.104, n = 6) **B and D**: The correlation of brain IL-6 (**B**) and TGF-β (**D**) mRNA expression with the sucrose preferences. Significant correlations were not observed.
