# Supplementary figures and images for: Sustained microglial activation in the area postrema of collagen-induced arthritis mice
Source: Arthritis Res Ther. 2021 Oct 29;23:273. doi: 10.1186/s13075-021-02657-x (PMC8556992; doi:10.1186/s13075-021-02657-x)

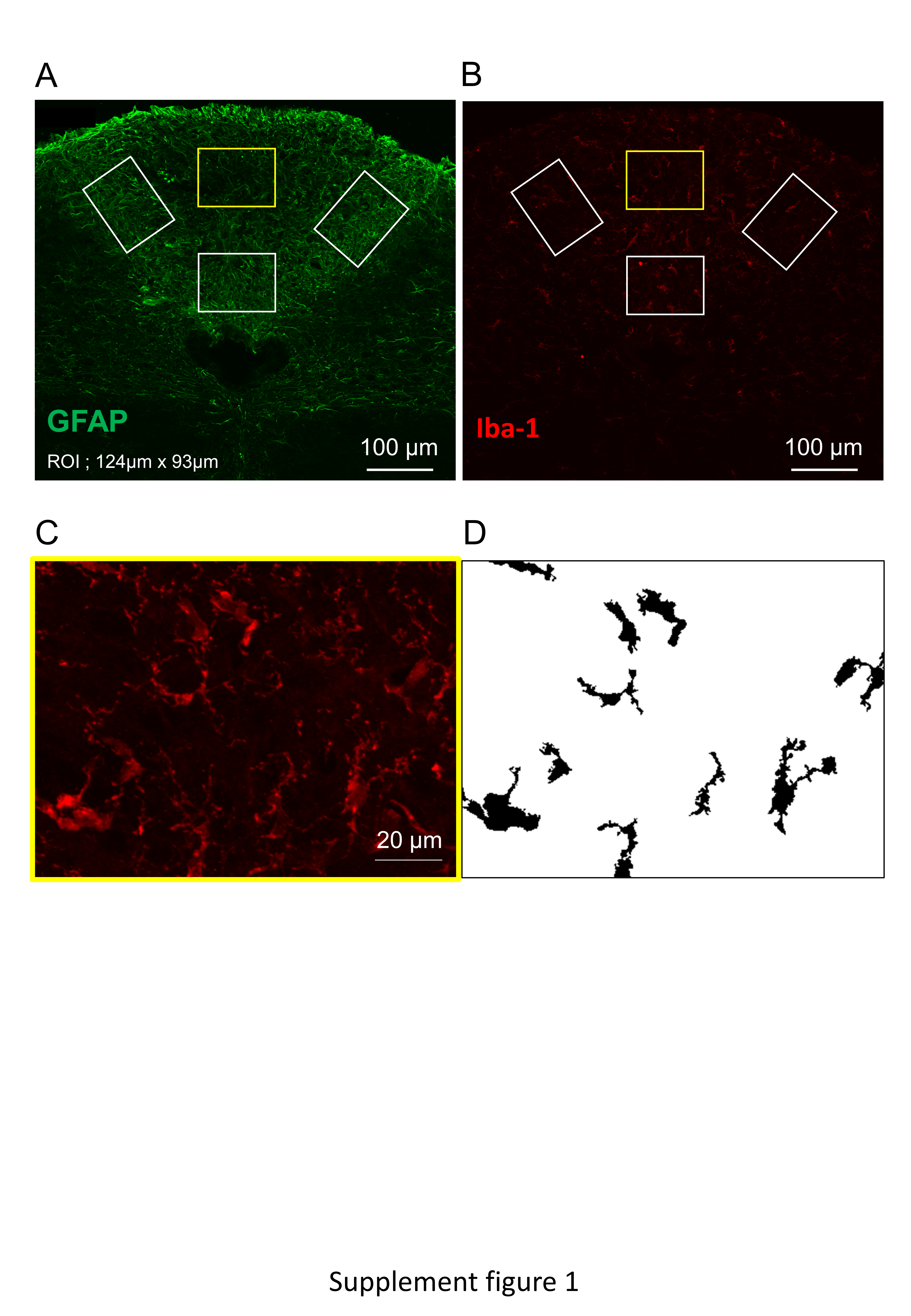

Supplement: Supplementary file 1 — Additional file 1: Supplementary Table 1. Component loading and variances of principal component analysis. PC-1, first principal component; PC-2, secondary principal component. Supplementary Figure 1. Four regions of interest (ROIs) for morphological analysis. A: Representative image showing the location of ROIs (124 μm × 93 μm, yellow and white boxes). ROIs were placed on the four main divisions described in previous reports [23, 24]. A blinded examiner placed ROIs by referring to immunostaining of glial fibrillary acidic protein (GFAP). B: ROIs on the image of immunostaining of ionized calcium-binding adaptor protein-1 (Iba-1). C: Higher magnification image of yellow boxed area in A and B. D: Binary image of C. Iba-1-staining was transformed to binary images using the “triangle methods”. Supplementary Figure 2. Representative examples of twelve measured morphological parameters. Binary images of ionized calcium-binding adaptor protein-1 (Iba-1) staining, like Supplementary Figure 1D, were used for the analysis. Area (μm2), perimeter length (μm), and circularity were measured using the outer edge (indicated by the red line). Major diameter, minimum diameter, aspect ratio, and roundness were measured using the best fitting ellipse (indicated by the blue line). The purple line shows the Feret diameter. Solidity was calculated using the convex hull (indicated by the green line). Width and height were measured using the bounding rectangle (indicated by the orange line). PID, post-immunization day. Supplementary Figure 3. Detection of sensory circumventricular organs (sCVOs) in DBA/1J mice. A: Illustration showing the general location of three sCVOs (indicated in green) in mouse brain. B: Upper panels show extravascular leakage of fluorescein isothiocyanate (FITC) in sCVOs of naïve DBA/1J mouse. After transcardial perfusion of FITC, fluorescence was diffusely observed in three regions adjacent to the ventricles. The lower panels show CD31 immunoreactivity in sCVOs. Immu [file 13075_2021_2657_MOESM1_ESM.zip › Figure S1.TIF]

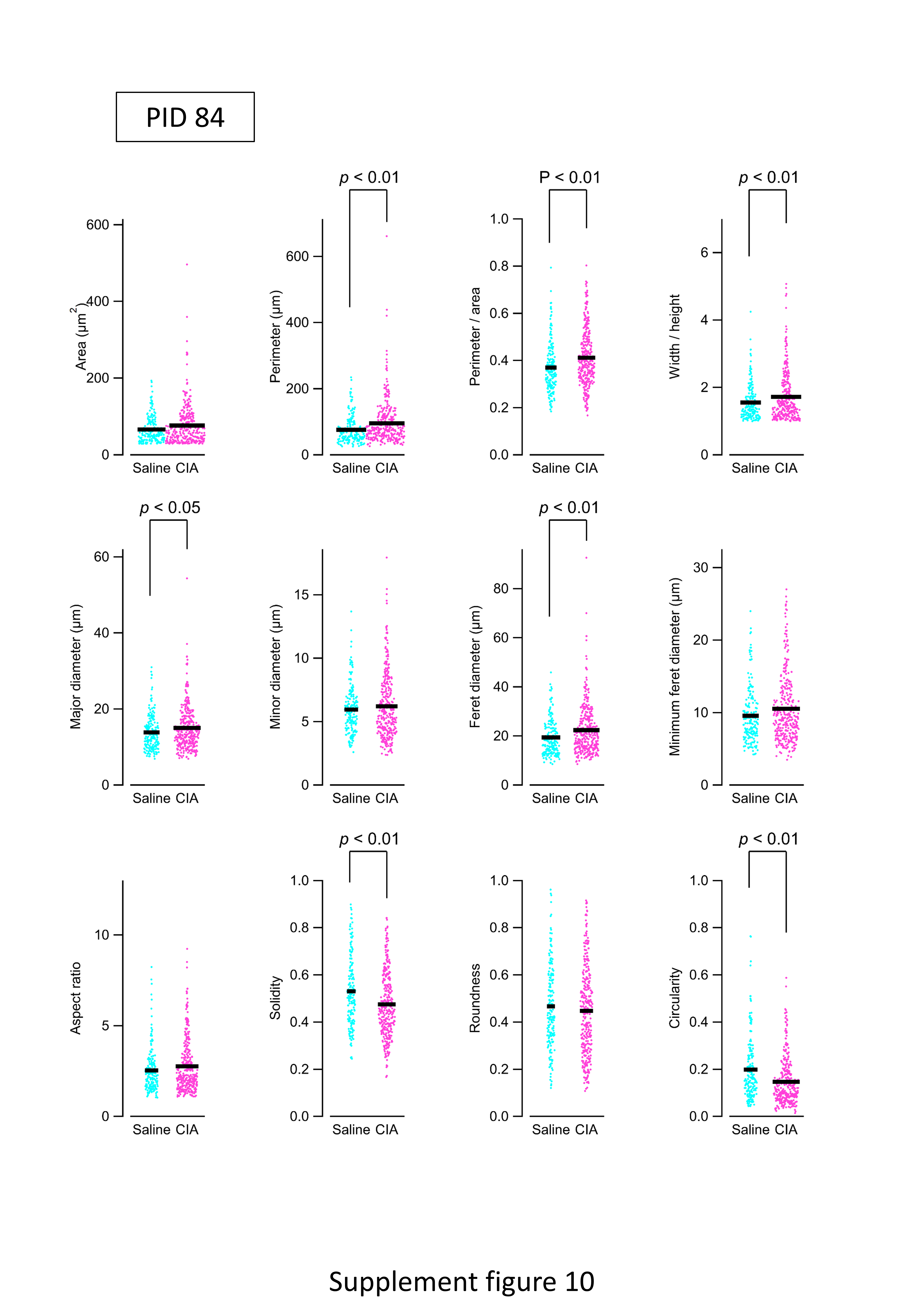

Supplement: Supplementary file 1 — Additional file 1: Supplementary Table 1. Component loading and variances of principal component analysis. PC-1, first principal component; PC-2, secondary principal component. Supplementary Figure 1. Four regions of interest (ROIs) for morphological analysis. A: Representative image showing the location of ROIs (124 μm × 93 μm, yellow and white boxes). ROIs were placed on the four main divisions described in previous reports [23, 24]. A blinded examiner placed ROIs by referring to immunostaining of glial fibrillary acidic protein (GFAP). B: ROIs on the image of immunostaining of ionized calcium-binding adaptor protein-1 (Iba-1). C: Higher magnification image of yellow boxed area in A and B. D: Binary image of C. Iba-1-staining was transformed to binary images using the “triangle methods”. Supplementary Figure 2. Representative examples of twelve measured morphological parameters. Binary images of ionized calcium-binding adaptor protein-1 (Iba-1) staining, like Supplementary Figure 1D, were used for the analysis. Area (μm2), perimeter length (μm), and circularity were measured using the outer edge (indicated by the red line). Major diameter, minimum diameter, aspect ratio, and roundness were measured using the best fitting ellipse (indicated by the blue line). The purple line shows the Feret diameter. Solidity was calculated using the convex hull (indicated by the green line). Width and height were measured using the bounding rectangle (indicated by the orange line). PID, post-immunization day. Supplementary Figure 3. Detection of sensory circumventricular organs (sCVOs) in DBA/1J mice. A: Illustration showing the general location of three sCVOs (indicated in green) in mouse brain. B: Upper panels show extravascular leakage of fluorescein isothiocyanate (FITC) in sCVOs of naïve DBA/1J mouse. After transcardial perfusion of FITC, fluorescence was diffusely observed in three regions adjacent to the ventricles. The lower panels show CD31 immunoreactivity in sCVOs. Immu [file 13075_2021_2657_MOESM1_ESM.zip › Figure S10.TIF]

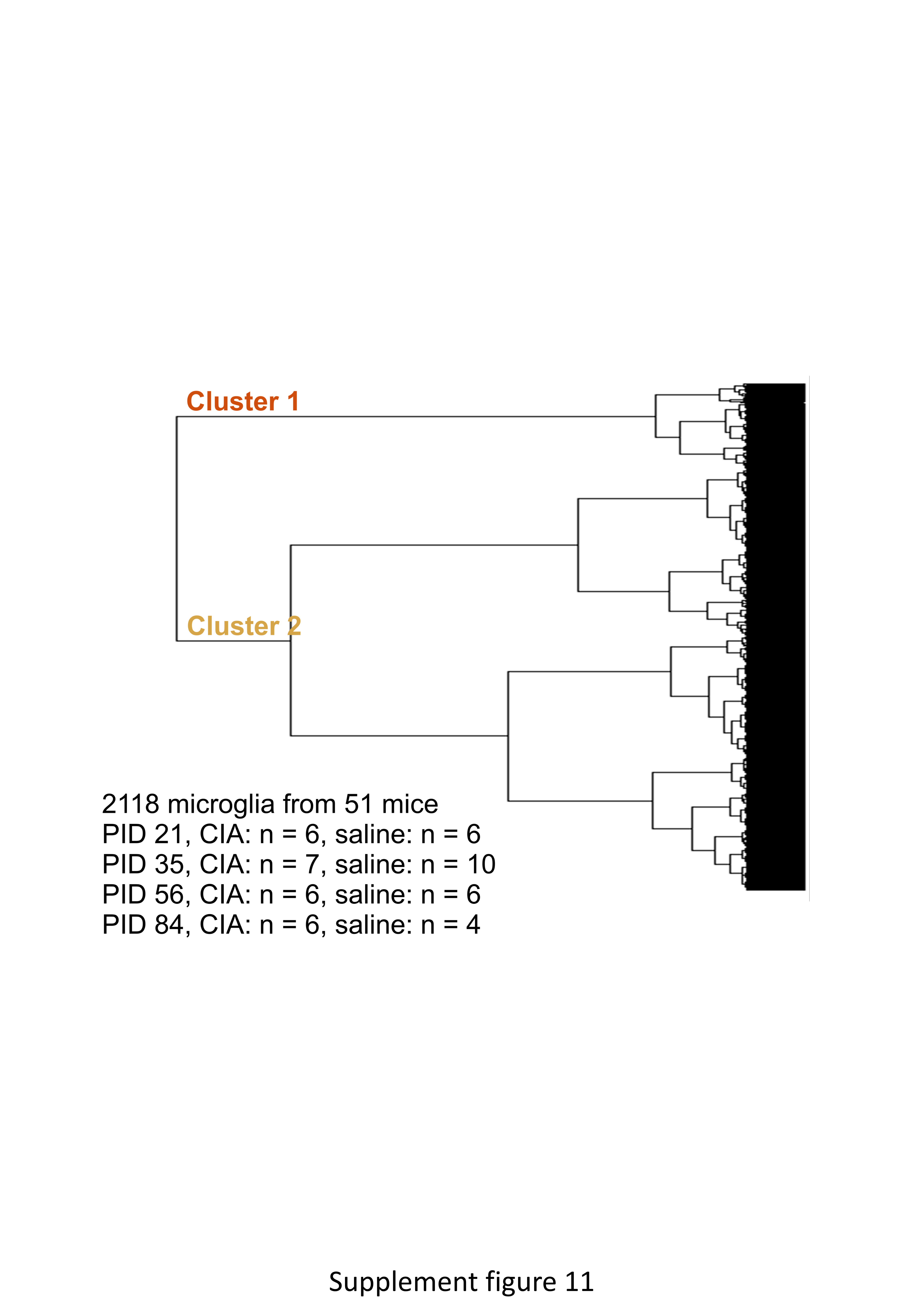

Supplement: Supplementary file 1 — Additional file 1: Supplementary Table 1. Component loading and variances of principal component analysis. PC-1, first principal component; PC-2, secondary principal component. Supplementary Figure 1. Four regions of interest (ROIs) for morphological analysis. A: Representative image showing the location of ROIs (124 μm × 93 μm, yellow and white boxes). ROIs were placed on the four main divisions described in previous reports [23, 24]. A blinded examiner placed ROIs by referring to immunostaining of glial fibrillary acidic protein (GFAP). B: ROIs on the image of immunostaining of ionized calcium-binding adaptor protein-1 (Iba-1). C: Higher magnification image of yellow boxed area in A and B. D: Binary image of C. Iba-1-staining was transformed to binary images using the “triangle methods”. Supplementary Figure 2. Representative examples of twelve measured morphological parameters. Binary images of ionized calcium-binding adaptor protein-1 (Iba-1) staining, like Supplementary Figure 1D, were used for the analysis. Area (μm2), perimeter length (μm), and circularity were measured using the outer edge (indicated by the red line). Major diameter, minimum diameter, aspect ratio, and roundness were measured using the best fitting ellipse (indicated by the blue line). The purple line shows the Feret diameter. Solidity was calculated using the convex hull (indicated by the green line). Width and height were measured using the bounding rectangle (indicated by the orange line). PID, post-immunization day. Supplementary Figure 3. Detection of sensory circumventricular organs (sCVOs) in DBA/1J mice. A: Illustration showing the general location of three sCVOs (indicated in green) in mouse brain. B: Upper panels show extravascular leakage of fluorescein isothiocyanate (FITC) in sCVOs of naïve DBA/1J mouse. After transcardial perfusion of FITC, fluorescence was diffusely observed in three regions adjacent to the ventricles. The lower panels show CD31 immunoreactivity in sCVOs. Immu [file 13075_2021_2657_MOESM1_ESM.zip › Figure S11.TIF]

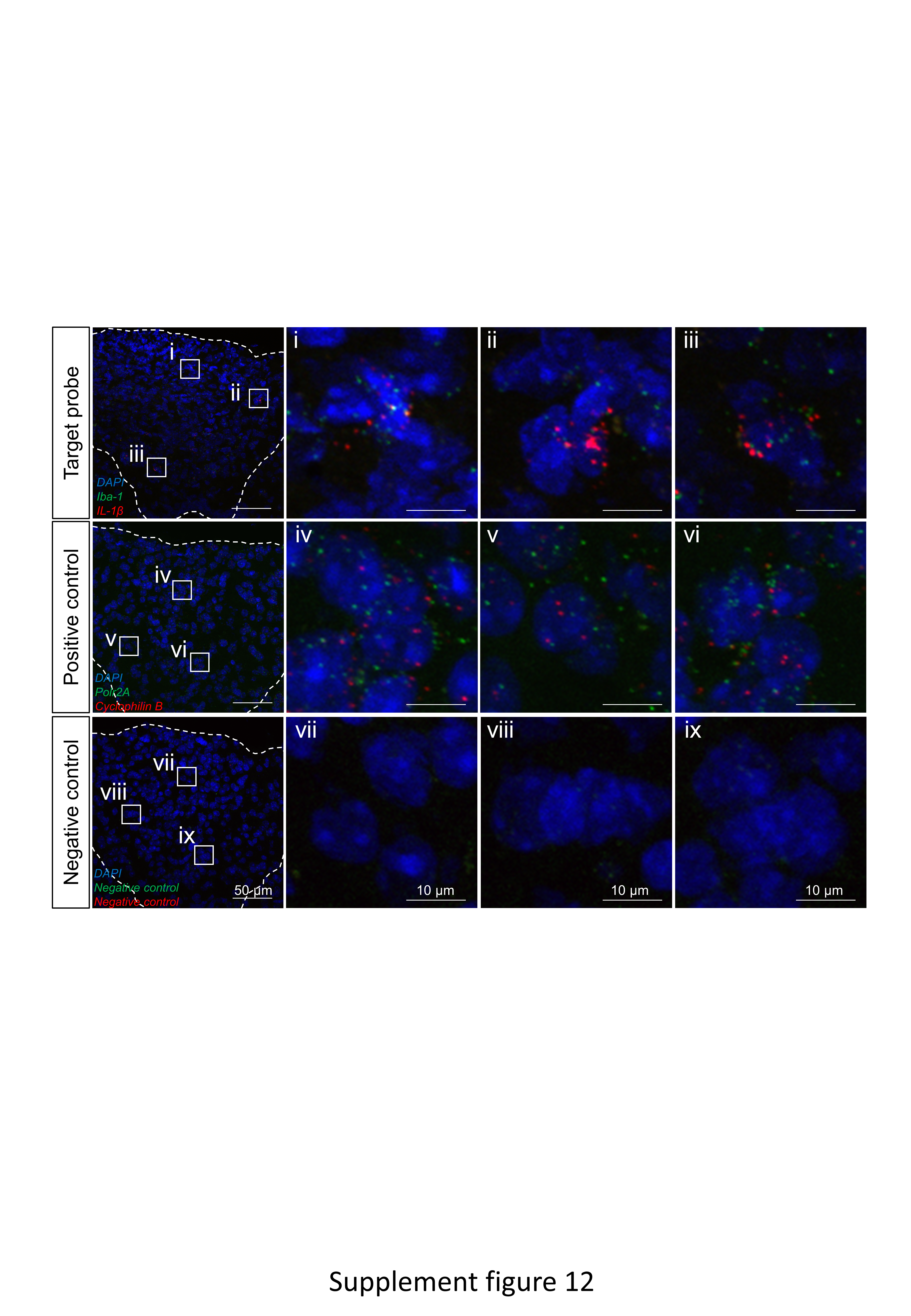

Supplement: Supplementary file 1 — Additional file 1: Supplementary Table 1. Component loading and variances of principal component analysis. PC-1, first principal component; PC-2, secondary principal component. Supplementary Figure 1. Four regions of interest (ROIs) for morphological analysis. A: Representative image showing the location of ROIs (124 μm × 93 μm, yellow and white boxes). ROIs were placed on the four main divisions described in previous reports [23, 24]. A blinded examiner placed ROIs by referring to immunostaining of glial fibrillary acidic protein (GFAP). B: ROIs on the image of immunostaining of ionized calcium-binding adaptor protein-1 (Iba-1). C: Higher magnification image of yellow boxed area in A and B. D: Binary image of C. Iba-1-staining was transformed to binary images using the “triangle methods”. Supplementary Figure 2. Representative examples of twelve measured morphological parameters. Binary images of ionized calcium-binding adaptor protein-1 (Iba-1) staining, like Supplementary Figure 1D, were used for the analysis. Area (μm2), perimeter length (μm), and circularity were measured using the outer edge (indicated by the red line). Major diameter, minimum diameter, aspect ratio, and roundness were measured using the best fitting ellipse (indicated by the blue line). The purple line shows the Feret diameter. Solidity was calculated using the convex hull (indicated by the green line). Width and height were measured using the bounding rectangle (indicated by the orange line). PID, post-immunization day. Supplementary Figure 3. Detection of sensory circumventricular organs (sCVOs) in DBA/1J mice. A: Illustration showing the general location of three sCVOs (indicated in green) in mouse brain. B: Upper panels show extravascular leakage of fluorescein isothiocyanate (FITC) in sCVOs of naïve DBA/1J mouse. After transcardial perfusion of FITC, fluorescence was diffusely observed in three regions adjacent to the ventricles. The lower panels show CD31 immunoreactivity in sCVOs. Immu [file 13075_2021_2657_MOESM1_ESM.zip › Figure S12.TIF]

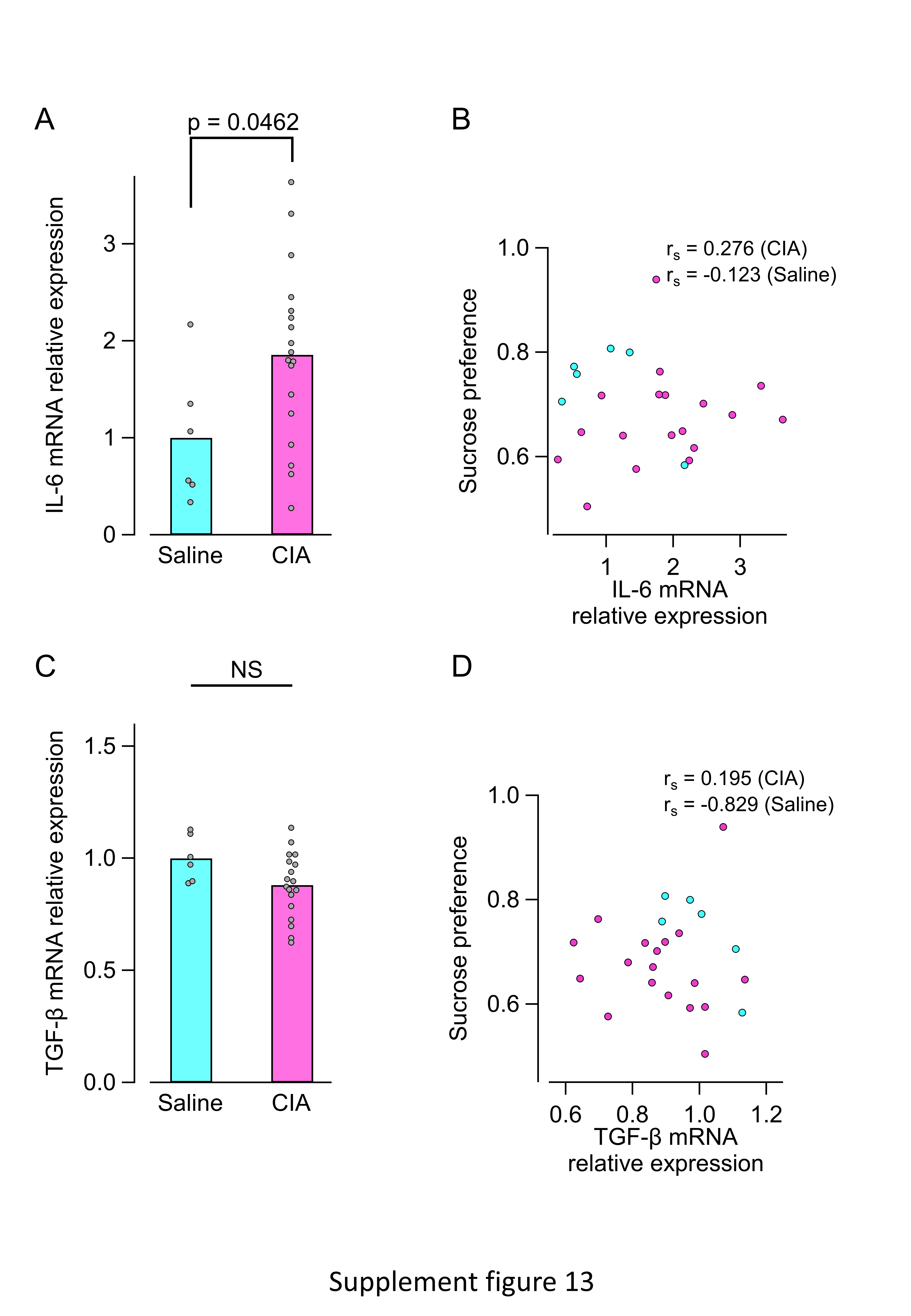

Supplement: Supplementary file 1 — Additional file 1: Supplementary Table 1. Component loading and variances of principal component analysis. PC-1, first principal component; PC-2, secondary principal component. Supplementary Figure 1. Four regions of interest (ROIs) for morphological analysis. A: Representative image showing the location of ROIs (124 μm × 93 μm, yellow and white boxes). ROIs were placed on the four main divisions described in previous reports [23, 24]. A blinded examiner placed ROIs by referring to immunostaining of glial fibrillary acidic protein (GFAP). B: ROIs on the image of immunostaining of ionized calcium-binding adaptor protein-1 (Iba-1). C: Higher magnification image of yellow boxed area in A and B. D: Binary image of C. Iba-1-staining was transformed to binary images using the “triangle methods”. Supplementary Figure 2. Representative examples of twelve measured morphological parameters. Binary images of ionized calcium-binding adaptor protein-1 (Iba-1) staining, like Supplementary Figure 1D, were used for the analysis. Area (μm2), perimeter length (μm), and circularity were measured using the outer edge (indicated by the red line). Major diameter, minimum diameter, aspect ratio, and roundness were measured using the best fitting ellipse (indicated by the blue line). The purple line shows the Feret diameter. Solidity was calculated using the convex hull (indicated by the green line). Width and height were measured using the bounding rectangle (indicated by the orange line). PID, post-immunization day. Supplementary Figure 3. Detection of sensory circumventricular organs (sCVOs) in DBA/1J mice. A: Illustration showing the general location of three sCVOs (indicated in green) in mouse brain. B: Upper panels show extravascular leakage of fluorescein isothiocyanate (FITC) in sCVOs of naïve DBA/1J mouse. After transcardial perfusion of FITC, fluorescence was diffusely observed in three regions adjacent to the ventricles. The lower panels show CD31 immunoreactivity in sCVOs. Immu [file 13075_2021_2657_MOESM1_ESM.zip › Figure S13.TIF]

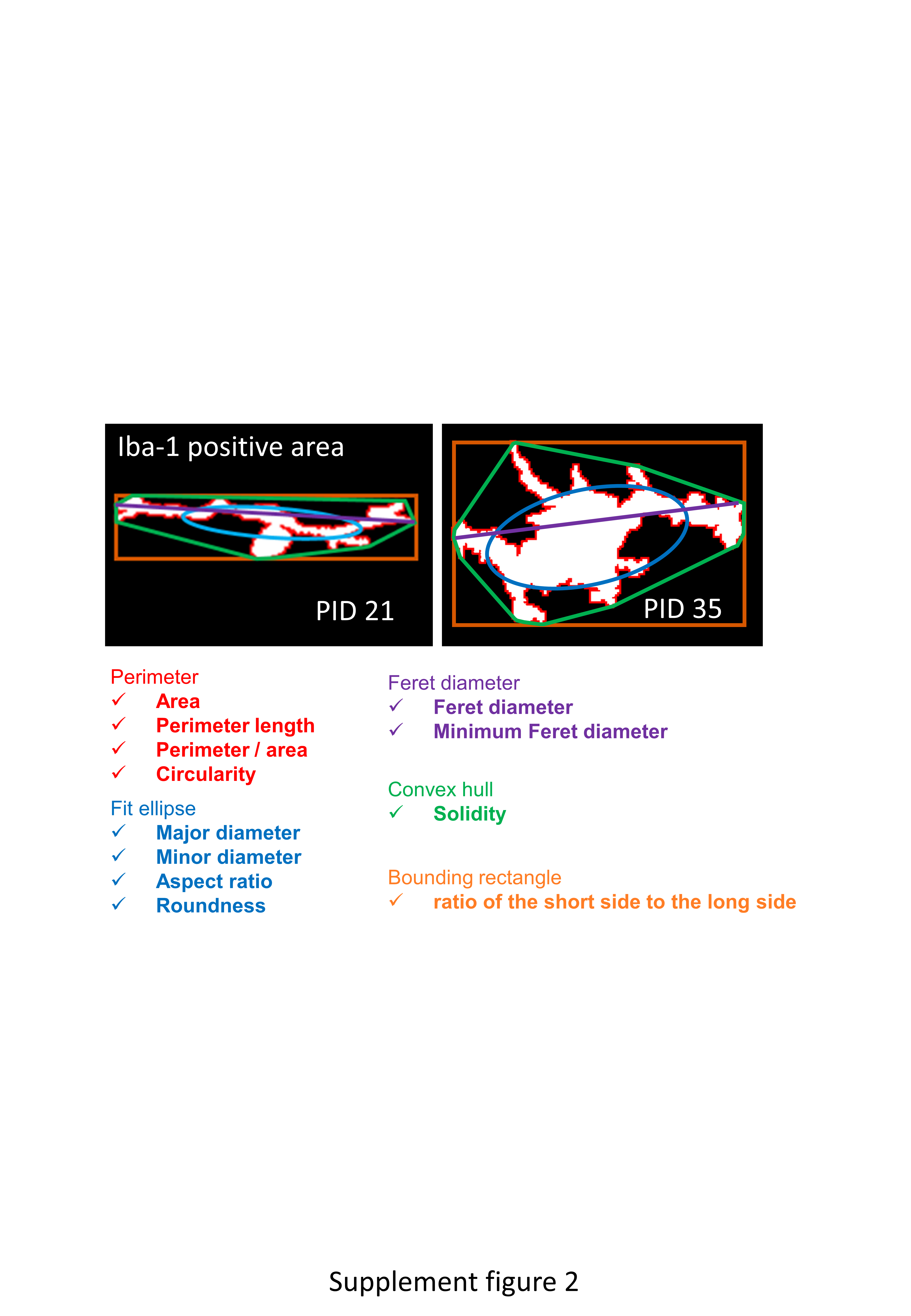

Supplement: Supplementary file 1 — Additional file 1: Supplementary Table 1. Component loading and variances of principal component analysis. PC-1, first principal component; PC-2, secondary principal component. Supplementary Figure 1. Four regions of interest (ROIs) for morphological analysis. A: Representative image showing the location of ROIs (124 μm × 93 μm, yellow and white boxes). ROIs were placed on the four main divisions described in previous reports [23, 24]. A blinded examiner placed ROIs by referring to immunostaining of glial fibrillary acidic protein (GFAP). B: ROIs on the image of immunostaining of ionized calcium-binding adaptor protein-1 (Iba-1). C: Higher magnification image of yellow boxed area in A and B. D: Binary image of C. Iba-1-staining was transformed to binary images using the “triangle methods”. Supplementary Figure 2. Representative examples of twelve measured morphological parameters. Binary images of ionized calcium-binding adaptor protein-1 (Iba-1) staining, like Supplementary Figure 1D, were used for the analysis. Area (μm2), perimeter length (μm), and circularity were measured using the outer edge (indicated by the red line). Major diameter, minimum diameter, aspect ratio, and roundness were measured using the best fitting ellipse (indicated by the blue line). The purple line shows the Feret diameter. Solidity was calculated using the convex hull (indicated by the green line). Width and height were measured using the bounding rectangle (indicated by the orange line). PID, post-immunization day. Supplementary Figure 3. Detection of sensory circumventricular organs (sCVOs) in DBA/1J mice. A: Illustration showing the general location of three sCVOs (indicated in green) in mouse brain. B: Upper panels show extravascular leakage of fluorescein isothiocyanate (FITC) in sCVOs of naïve DBA/1J mouse. After transcardial perfusion of FITC, fluorescence was diffusely observed in three regions adjacent to the ventricles. The lower panels show CD31 immunoreactivity in sCVOs. Immu [file 13075_2021_2657_MOESM1_ESM.zip › Figure S2.TIF]

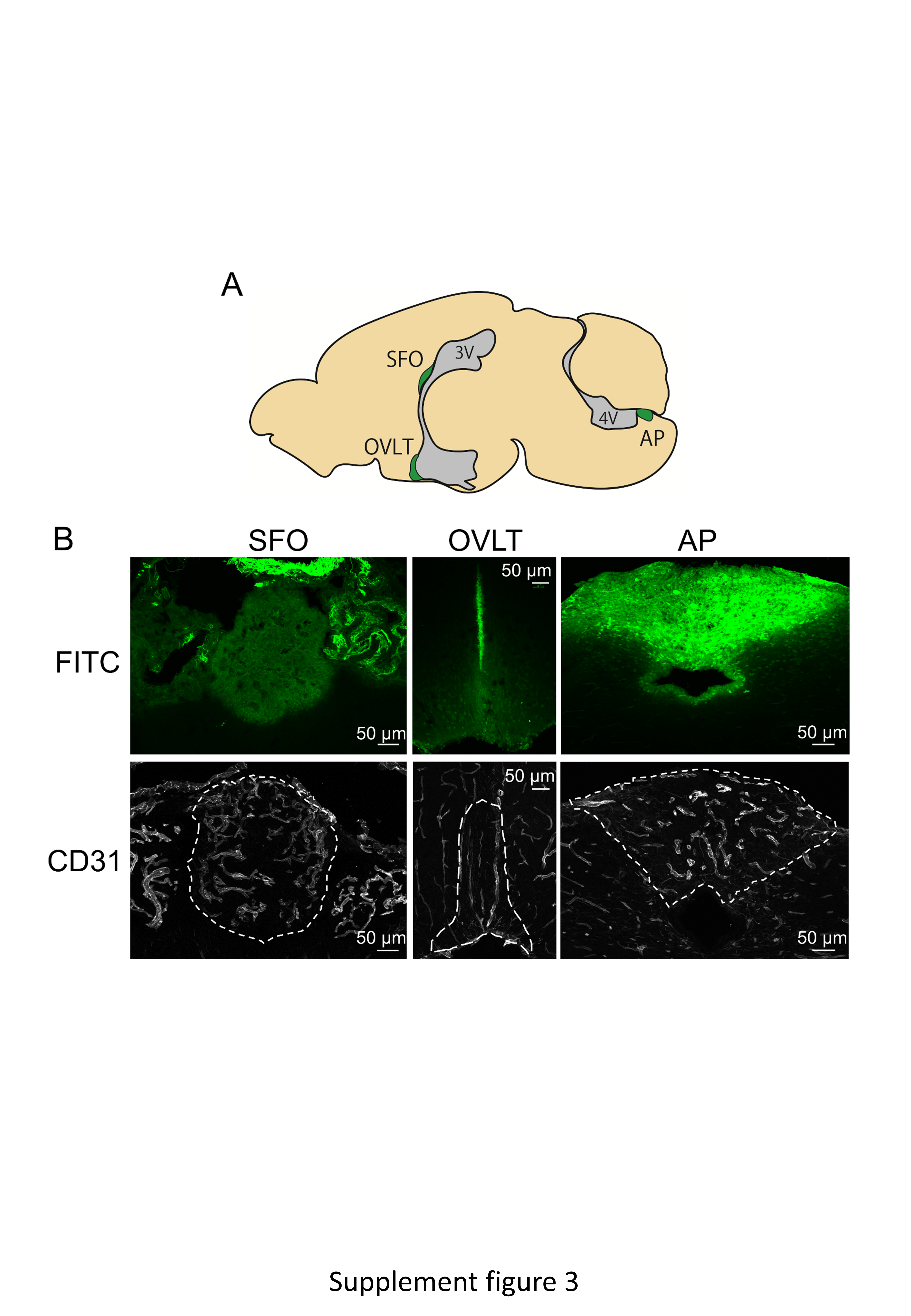

Supplement: Supplementary file 1 — Additional file 1: Supplementary Table 1. Component loading and variances of principal component analysis. PC-1, first principal component; PC-2, secondary principal component. Supplementary Figure 1. Four regions of interest (ROIs) for morphological analysis. A: Representative image showing the location of ROIs (124 μm × 93 μm, yellow and white boxes). ROIs were placed on the four main divisions described in previous reports [23, 24]. A blinded examiner placed ROIs by referring to immunostaining of glial fibrillary acidic protein (GFAP). B: ROIs on the image of immunostaining of ionized calcium-binding adaptor protein-1 (Iba-1). C: Higher magnification image of yellow boxed area in A and B. D: Binary image of C. Iba-1-staining was transformed to binary images using the “triangle methods”. Supplementary Figure 2. Representative examples of twelve measured morphological parameters. Binary images of ionized calcium-binding adaptor protein-1 (Iba-1) staining, like Supplementary Figure 1D, were used for the analysis. Area (μm2), perimeter length (μm), and circularity were measured using the outer edge (indicated by the red line). Major diameter, minimum diameter, aspect ratio, and roundness were measured using the best fitting ellipse (indicated by the blue line). The purple line shows the Feret diameter. Solidity was calculated using the convex hull (indicated by the green line). Width and height were measured using the bounding rectangle (indicated by the orange line). PID, post-immunization day. Supplementary Figure 3. Detection of sensory circumventricular organs (sCVOs) in DBA/1J mice. A: Illustration showing the general location of three sCVOs (indicated in green) in mouse brain. B: Upper panels show extravascular leakage of fluorescein isothiocyanate (FITC) in sCVOs of naïve DBA/1J mouse. After transcardial perfusion of FITC, fluorescence was diffusely observed in three regions adjacent to the ventricles. The lower panels show CD31 immunoreactivity in sCVOs. Immu [file 13075_2021_2657_MOESM1_ESM.zip › Figure S3.TIF]

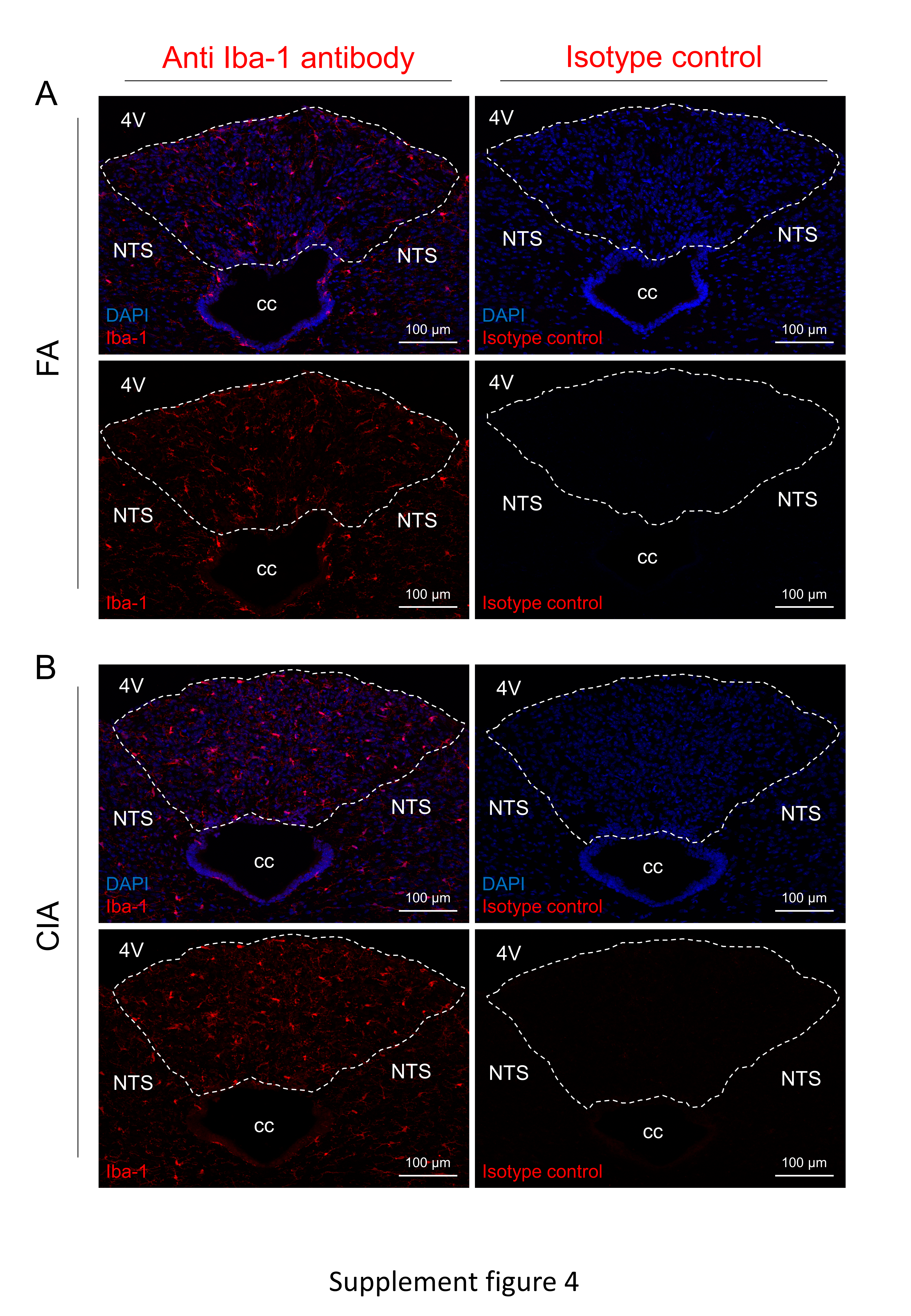

Supplement: Supplementary file 1 — Additional file 1: Supplementary Table 1. Component loading and variances of principal component analysis. PC-1, first principal component; PC-2, secondary principal component. Supplementary Figure 1. Four regions of interest (ROIs) for morphological analysis. A: Representative image showing the location of ROIs (124 μm × 93 μm, yellow and white boxes). ROIs were placed on the four main divisions described in previous reports [23, 24]. A blinded examiner placed ROIs by referring to immunostaining of glial fibrillary acidic protein (GFAP). B: ROIs on the image of immunostaining of ionized calcium-binding adaptor protein-1 (Iba-1). C: Higher magnification image of yellow boxed area in A and B. D: Binary image of C. Iba-1-staining was transformed to binary images using the “triangle methods”. Supplementary Figure 2. Representative examples of twelve measured morphological parameters. Binary images of ionized calcium-binding adaptor protein-1 (Iba-1) staining, like Supplementary Figure 1D, were used for the analysis. Area (μm2), perimeter length (μm), and circularity were measured using the outer edge (indicated by the red line). Major diameter, minimum diameter, aspect ratio, and roundness were measured using the best fitting ellipse (indicated by the blue line). The purple line shows the Feret diameter. Solidity was calculated using the convex hull (indicated by the green line). Width and height were measured using the bounding rectangle (indicated by the orange line). PID, post-immunization day. Supplementary Figure 3. Detection of sensory circumventricular organs (sCVOs) in DBA/1J mice. A: Illustration showing the general location of three sCVOs (indicated in green) in mouse brain. B: Upper panels show extravascular leakage of fluorescein isothiocyanate (FITC) in sCVOs of naïve DBA/1J mouse. After transcardial perfusion of FITC, fluorescence was diffusely observed in three regions adjacent to the ventricles. The lower panels show CD31 immunoreactivity in sCVOs. Immu [file 13075_2021_2657_MOESM1_ESM.zip › Figure S4.TIF]

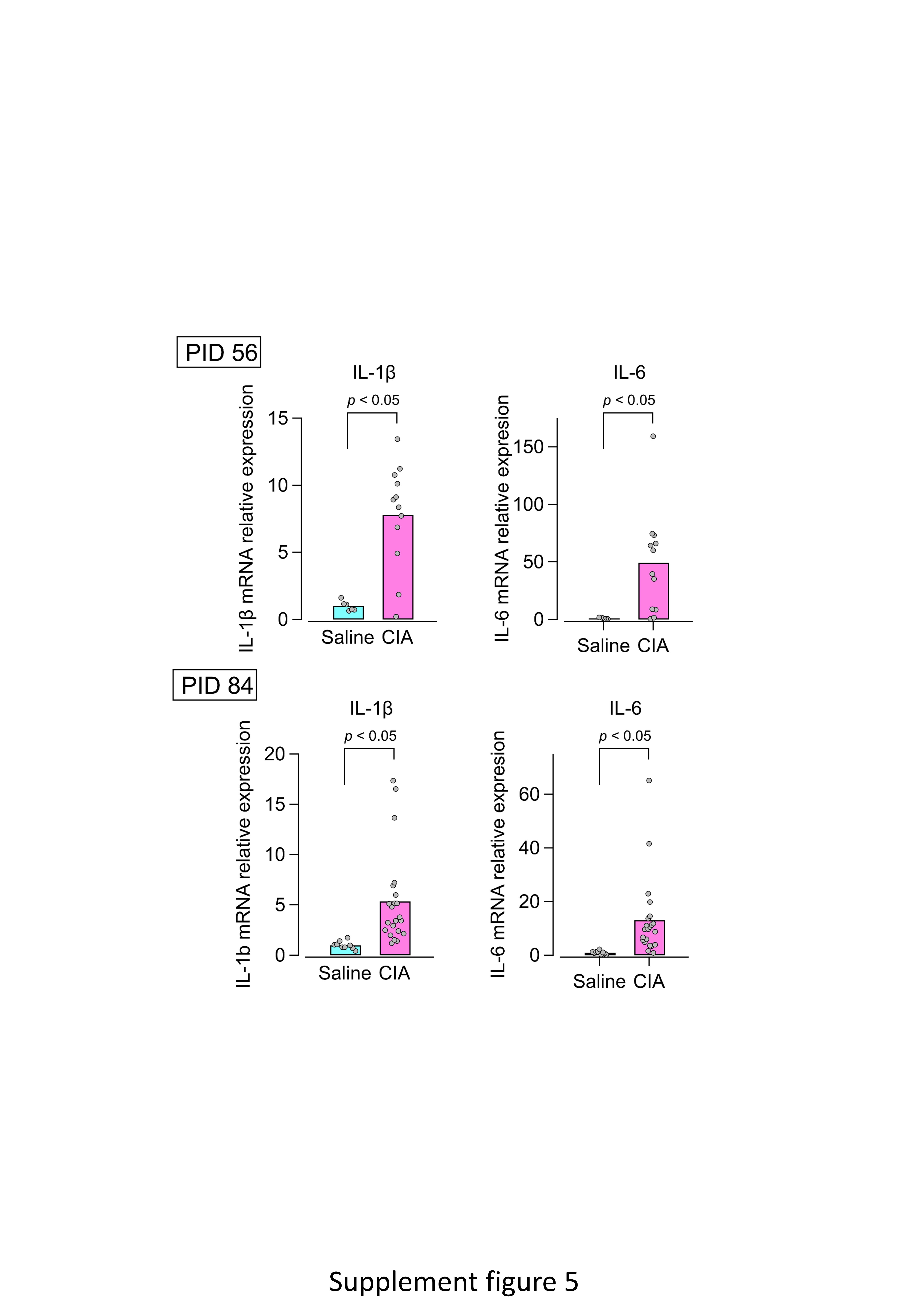

Supplement: Supplementary file 1 — Additional file 1: Supplementary Table 1. Component loading and variances of principal component analysis. PC-1, first principal component; PC-2, secondary principal component. Supplementary Figure 1. Four regions of interest (ROIs) for morphological analysis. A: Representative image showing the location of ROIs (124 μm × 93 μm, yellow and white boxes). ROIs were placed on the four main divisions described in previous reports [23, 24]. A blinded examiner placed ROIs by referring to immunostaining of glial fibrillary acidic protein (GFAP). B: ROIs on the image of immunostaining of ionized calcium-binding adaptor protein-1 (Iba-1). C: Higher magnification image of yellow boxed area in A and B. D: Binary image of C. Iba-1-staining was transformed to binary images using the “triangle methods”. Supplementary Figure 2. Representative examples of twelve measured morphological parameters. Binary images of ionized calcium-binding adaptor protein-1 (Iba-1) staining, like Supplementary Figure 1D, were used for the analysis. Area (μm2), perimeter length (μm), and circularity were measured using the outer edge (indicated by the red line). Major diameter, minimum diameter, aspect ratio, and roundness were measured using the best fitting ellipse (indicated by the blue line). The purple line shows the Feret diameter. Solidity was calculated using the convex hull (indicated by the green line). Width and height were measured using the bounding rectangle (indicated by the orange line). PID, post-immunization day. Supplementary Figure 3. Detection of sensory circumventricular organs (sCVOs) in DBA/1J mice. A: Illustration showing the general location of three sCVOs (indicated in green) in mouse brain. B: Upper panels show extravascular leakage of fluorescein isothiocyanate (FITC) in sCVOs of naïve DBA/1J mouse. After transcardial perfusion of FITC, fluorescence was diffusely observed in three regions adjacent to the ventricles. The lower panels show CD31 immunoreactivity in sCVOs. Immu [file 13075_2021_2657_MOESM1_ESM.zip › Figure S5.TIF]

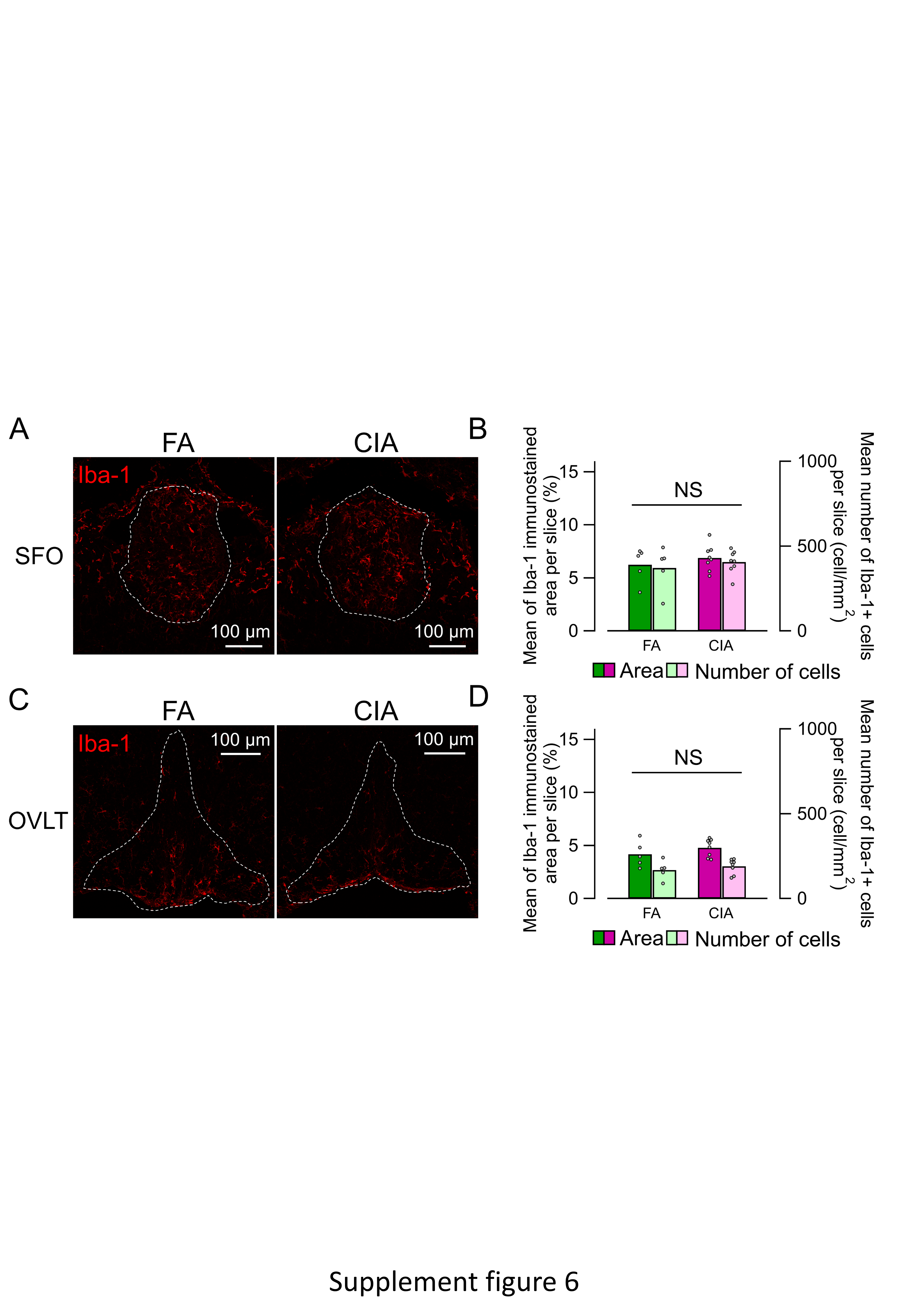

Supplement: Supplementary file 1 — Additional file 1: Supplementary Table 1. Component loading and variances of principal component analysis. PC-1, first principal component; PC-2, secondary principal component. Supplementary Figure 1. Four regions of interest (ROIs) for morphological analysis. A: Representative image showing the location of ROIs (124 μm × 93 μm, yellow and white boxes). ROIs were placed on the four main divisions described in previous reports [23, 24]. A blinded examiner placed ROIs by referring to immunostaining of glial fibrillary acidic protein (GFAP). B: ROIs on the image of immunostaining of ionized calcium-binding adaptor protein-1 (Iba-1). C: Higher magnification image of yellow boxed area in A and B. D: Binary image of C. Iba-1-staining was transformed to binary images using the “triangle methods”. Supplementary Figure 2. Representative examples of twelve measured morphological parameters. Binary images of ionized calcium-binding adaptor protein-1 (Iba-1) staining, like Supplementary Figure 1D, were used for the analysis. Area (μm2), perimeter length (μm), and circularity were measured using the outer edge (indicated by the red line). Major diameter, minimum diameter, aspect ratio, and roundness were measured using the best fitting ellipse (indicated by the blue line). The purple line shows the Feret diameter. Solidity was calculated using the convex hull (indicated by the green line). Width and height were measured using the bounding rectangle (indicated by the orange line). PID, post-immunization day. Supplementary Figure 3. Detection of sensory circumventricular organs (sCVOs) in DBA/1J mice. A: Illustration showing the general location of three sCVOs (indicated in green) in mouse brain. B: Upper panels show extravascular leakage of fluorescein isothiocyanate (FITC) in sCVOs of naïve DBA/1J mouse. After transcardial perfusion of FITC, fluorescence was diffusely observed in three regions adjacent to the ventricles. The lower panels show CD31 immunoreactivity in sCVOs. Immu [file 13075_2021_2657_MOESM1_ESM.zip › Figure S6.TIF]

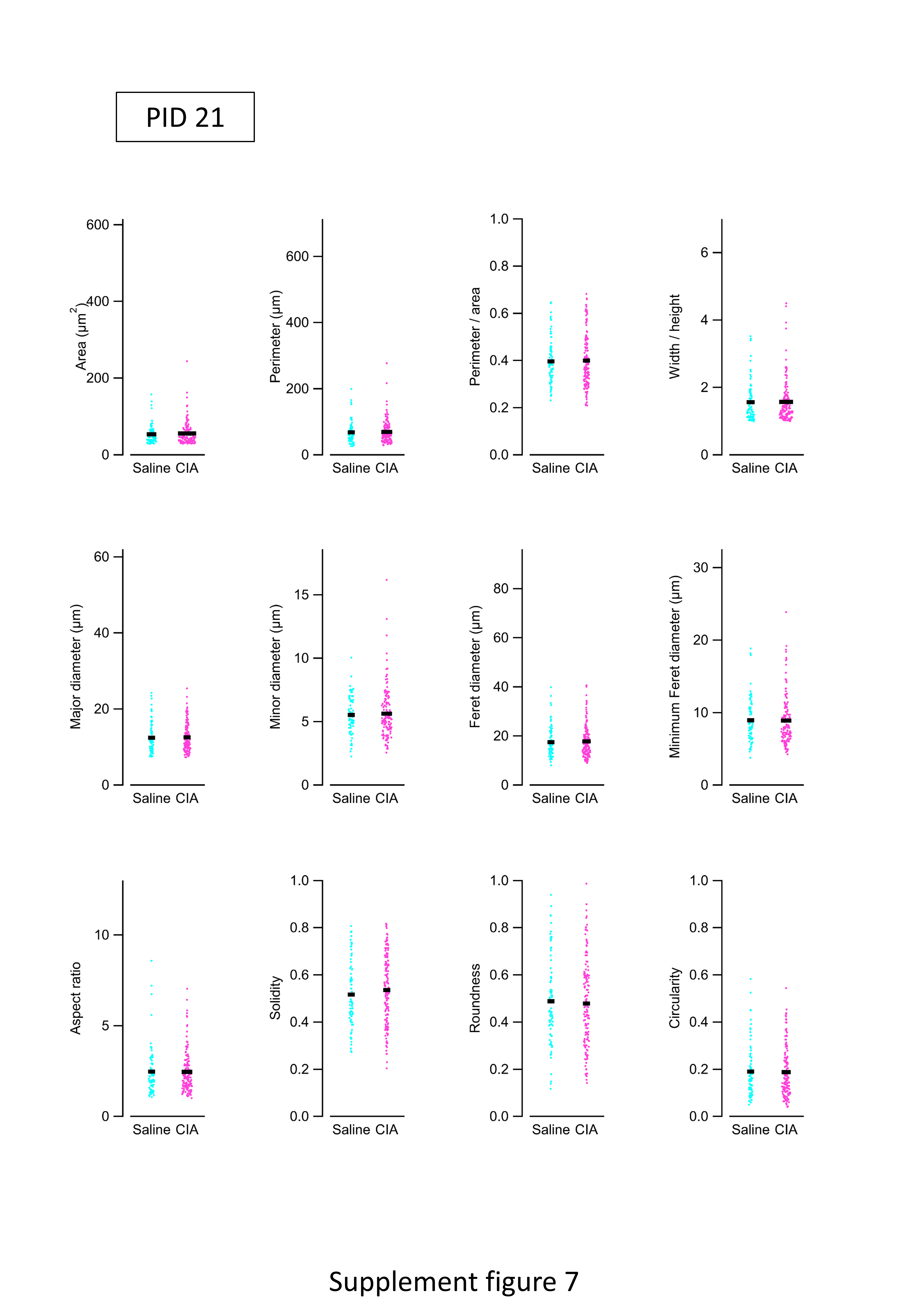

Supplement: Supplementary file 1 — Additional file 1: Supplementary Table 1. Component loading and variances of principal component analysis. PC-1, first principal component; PC-2, secondary principal component. Supplementary Figure 1. Four regions of interest (ROIs) for morphological analysis. A: Representative image showing the location of ROIs (124 μm × 93 μm, yellow and white boxes). ROIs were placed on the four main divisions described in previous reports [23, 24]. A blinded examiner placed ROIs by referring to immunostaining of glial fibrillary acidic protein (GFAP). B: ROIs on the image of immunostaining of ionized calcium-binding adaptor protein-1 (Iba-1). C: Higher magnification image of yellow boxed area in A and B. D: Binary image of C. Iba-1-staining was transformed to binary images using the “triangle methods”. Supplementary Figure 2. Representative examples of twelve measured morphological parameters. Binary images of ionized calcium-binding adaptor protein-1 (Iba-1) staining, like Supplementary Figure 1D, were used for the analysis. Area (μm2), perimeter length (μm), and circularity were measured using the outer edge (indicated by the red line). Major diameter, minimum diameter, aspect ratio, and roundness were measured using the best fitting ellipse (indicated by the blue line). The purple line shows the Feret diameter. Solidity was calculated using the convex hull (indicated by the green line). Width and height were measured using the bounding rectangle (indicated by the orange line). PID, post-immunization day. Supplementary Figure 3. Detection of sensory circumventricular organs (sCVOs) in DBA/1J mice. A: Illustration showing the general location of three sCVOs (indicated in green) in mouse brain. B: Upper panels show extravascular leakage of fluorescein isothiocyanate (FITC) in sCVOs of naïve DBA/1J mouse. After transcardial perfusion of FITC, fluorescence was diffusely observed in three regions adjacent to the ventricles. The lower panels show CD31 immunoreactivity in sCVOs. Immu [file 13075_2021_2657_MOESM1_ESM.zip › Figure S7.TIF]

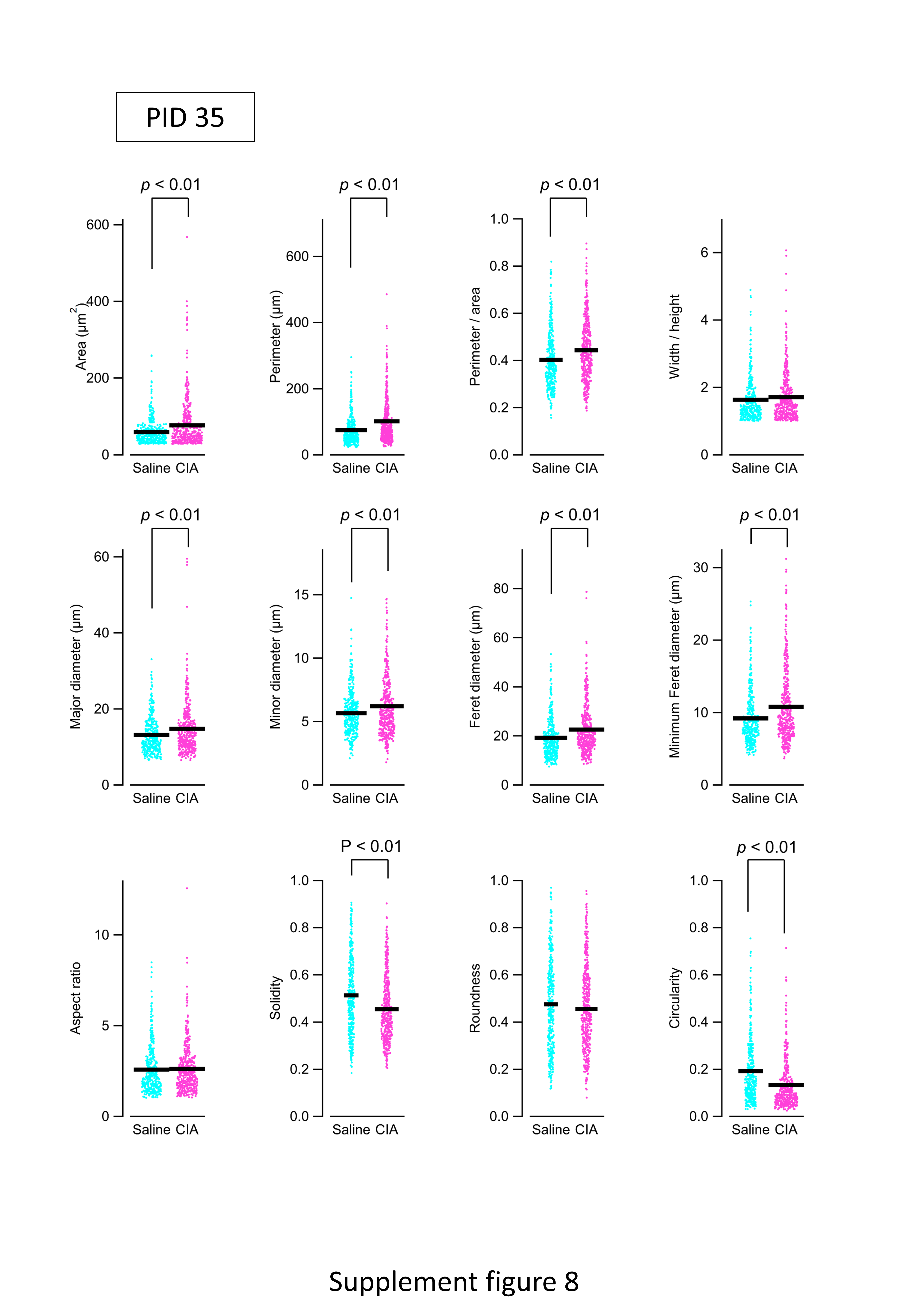

Supplement: Supplementary file 1 — Additional file 1: Supplementary Table 1. Component loading and variances of principal component analysis. PC-1, first principal component; PC-2, secondary principal component. Supplementary Figure 1. Four regions of interest (ROIs) for morphological analysis. A: Representative image showing the location of ROIs (124 μm × 93 μm, yellow and white boxes). ROIs were placed on the four main divisions described in previous reports [23, 24]. A blinded examiner placed ROIs by referring to immunostaining of glial fibrillary acidic protein (GFAP). B: ROIs on the image of immunostaining of ionized calcium-binding adaptor protein-1 (Iba-1). C: Higher magnification image of yellow boxed area in A and B. D: Binary image of C. Iba-1-staining was transformed to binary images using the “triangle methods”. Supplementary Figure 2. Representative examples of twelve measured morphological parameters. Binary images of ionized calcium-binding adaptor protein-1 (Iba-1) staining, like Supplementary Figure 1D, were used for the analysis. Area (μm2), perimeter length (μm), and circularity were measured using the outer edge (indicated by the red line). Major diameter, minimum diameter, aspect ratio, and roundness were measured using the best fitting ellipse (indicated by the blue line). The purple line shows the Feret diameter. Solidity was calculated using the convex hull (indicated by the green line). Width and height were measured using the bounding rectangle (indicated by the orange line). PID, post-immunization day. Supplementary Figure 3. Detection of sensory circumventricular organs (sCVOs) in DBA/1J mice. A: Illustration showing the general location of three sCVOs (indicated in green) in mouse brain. B: Upper panels show extravascular leakage of fluorescein isothiocyanate (FITC) in sCVOs of naïve DBA/1J mouse. After transcardial perfusion of FITC, fluorescence was diffusely observed in three regions adjacent to the ventricles. The lower panels show CD31 immunoreactivity in sCVOs. Immu [file 13075_2021_2657_MOESM1_ESM.zip › Figure S8.TIF]

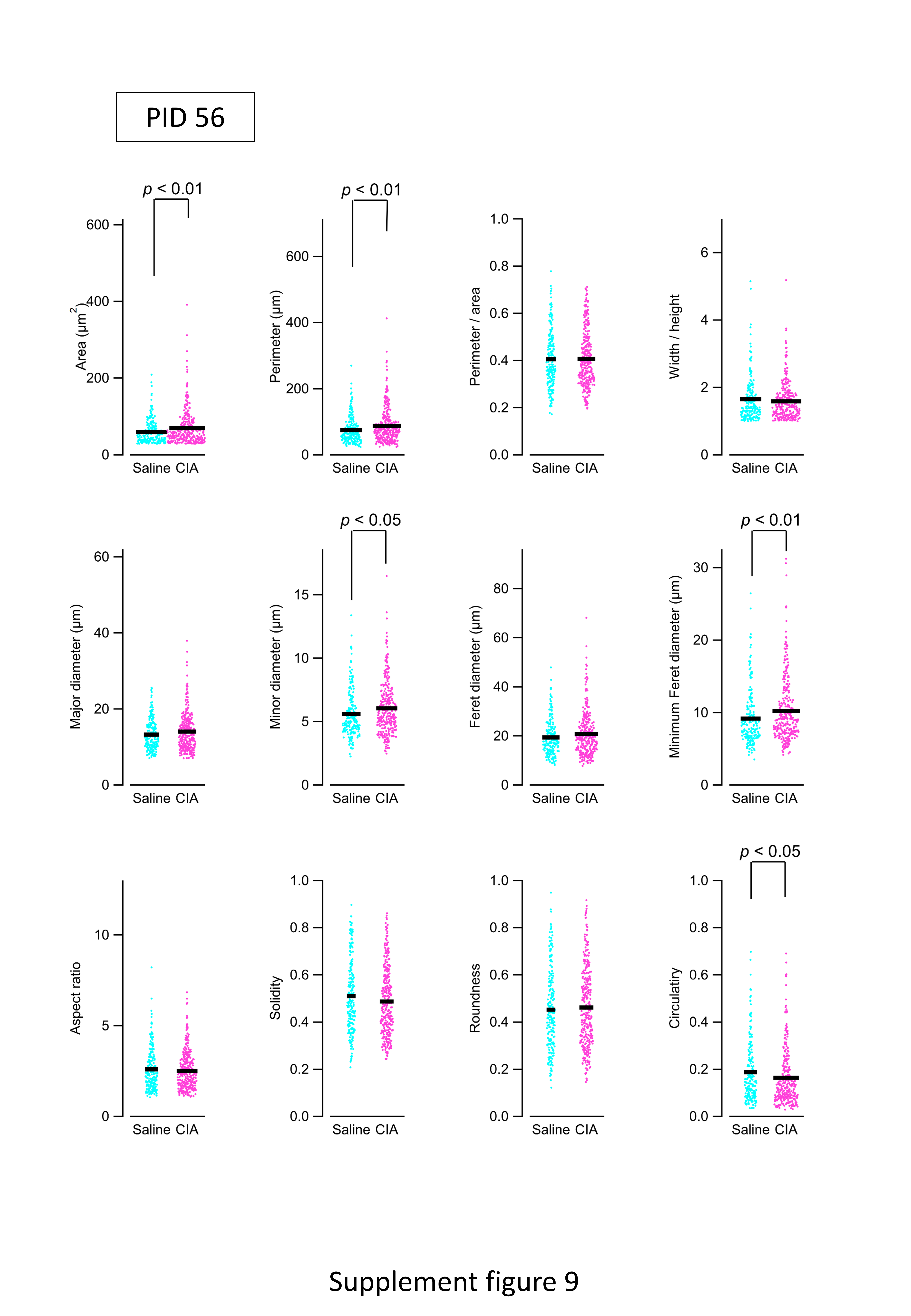

Supplement: Supplementary file 1 — Additional file 1: Supplementary Table 1. Component loading and variances of principal component analysis. PC-1, first principal component; PC-2, secondary principal component. Supplementary Figure 1. Four regions of interest (ROIs) for morphological analysis. A: Representative image showing the location of ROIs (124 μm × 93 μm, yellow and white boxes). ROIs were placed on the four main divisions described in previous reports [23, 24]. A blinded examiner placed ROIs by referring to immunostaining of glial fibrillary acidic protein (GFAP). B: ROIs on the image of immunostaining of ionized calcium-binding adaptor protein-1 (Iba-1). C: Higher magnification image of yellow boxed area in A and B. D: Binary image of C. Iba-1-staining was transformed to binary images using the “triangle methods”. Supplementary Figure 2. Representative examples of twelve measured morphological parameters. Binary images of ionized calcium-binding adaptor protein-1 (Iba-1) staining, like Supplementary Figure 1D, were used for the analysis. Area (μm2), perimeter length (μm), and circularity were measured using the outer edge (indicated by the red line). Major diameter, minimum diameter, aspect ratio, and roundness were measured using the best fitting ellipse (indicated by the blue line). The purple line shows the Feret diameter. Solidity was calculated using the convex hull (indicated by the green line). Width and height were measured using the bounding rectangle (indicated by the orange line). PID, post-immunization day. Supplementary Figure 3. Detection of sensory circumventricular organs (sCVOs) in DBA/1J mice. A: Illustration showing the general location of three sCVOs (indicated in green) in mouse brain. B: Upper panels show extravascular leakage of fluorescein isothiocyanate (FITC) in sCVOs of naïve DBA/1J mouse. After transcardial perfusion of FITC, fluorescence was diffusely observed in three regions adjacent to the ventricles. The lower panels show CD31 immunoreactivity in sCVOs. Immu [file 13075_2021_2657_MOESM1_ESM.zip › Figure S9.TIF]
